# Syntheses, crystal structures and Hirshfeld surface analysis of three salts of 1-(4-nitro­phenyl)­piperazine

**DOI:** 10.1107/S2056989023002517

**Published:** 2023-03-21

**Authors:** Sreeramapura D. Archana, Sabine Foro, Hemmige S. Yathirajan, Haruvegowda Kiran Kumar, Rishik Balerao, Ray J. Butcher

**Affiliations:** a Department of Studies in Chemistry, University of Mysore, Manasagangotri, Mysore-570 006, India; b Institute of Materials Science, Darmstadt University of Technology, Alarich-Weiss-Strasse 2, D-64287 Darmstadt, Germany; c Thomas Jefferson High School for Science and Technology, 6560 Braddock Rd, Alexandria VA 22312, USA; d Department of Chemistry, Howard University, 525 College Street NW, Washington DC 20059, USA; Katholieke Universiteit Leuven, Belgium

**Keywords:** crystal structure, piperazinium salt, Hirshfeld surface analysis, salicylate, graph-set notation, axial and equatorial substitution patterns in piperazinium rings

## Abstract

The structures and Hirshfeld surface analysis of three salts of 1-(4-nitro­phenyl)­piperazine are discussed. In 4-(4-nitro­phen­yl)piperazin-1-ium salicylate (C_10_H_14_N_3_O_2_^+^·C_7_H_5_O_3_^−^), there are strong hydrogen bonds between cation and anion and the 4-nitro­phenyl substituent occupies an equatorial position in the piperazinium ring. The cation and anion are linked together by supra­molecular inter­actions [graph-set notation of hydrogen bonding  (6) propagating in the *a*-axis direction]. Additionally, there is π–π stacking involving the salicylate anion and the piperazinium cation in adjacent asymmetric units as well as a C—H⋯π inter­action between a hydrogen atom on the piperazine ring and the phenyl ring within the salicyclate anion. In bis­[4-(4-nitro­phen­yl)piperazin-1-ium] bis­(4-fluoro­benzoate) trihydrate (2C_10_H_14_N_3_O_2_^+^·2C_7_H_4_FO_2_^−^·3H_2_O), there are two cations, two anions, and three water mol­ecules of solvation in the asymmetric unit, all linked by hydrogen bonds [graph-set notation of hydrogen bonding *R* ^2^_2_(20) between adjacent cations and *R* ^3^_3_(9) between a cation and its adjacent anion]. In the anion, the 4-nitro­phenyl ring occupies an axial substitution position in the piperazinium ring, which is relatively rare. Within the asymmetric unit, the phenyl groups in the cations show an offset π–π inter­action. Additionally, there is a C—H⋯π inter­action between a hydrogen atom on the phenyl ring within a cation and the phenyl ring within an anion. In 4-(4-nitro­phen­yl)piperazin-1-ium 3,5-di­nitro­benzoate (C_10_H_14_N_3_O_2_^+^·C_7_H_4_N_2_O_6_^−^), there is a strong N—H⋯O hydrogen bond linking the cation and anion and the 4-nitro­phenyl ring occupies an axial substitution position in the piperazinium ring, as seen in the previous structure. In the crystal, the cation and the anion form a complex three-dimensional hydrogen-bonded array involving *R* ^2^_2_(8), *R* ^4^_4_(12) and *R* ^4^_4_(20) rings propogating in the *a*-axis direction. The nitro­phenyl group is disordered with occupancies of 0.806 (10) and 0.194 (10).

## Abstract 2

Three salts of 4-nitro­phenyl­piperazine were synthesized and their crystal structures determined *via* X-ray diffraction.

## 1. Chemical context

Piperazines and substituted piperazines are important pharmacophores that can be found in many biologically active compounds across a number of different therapeutic areas (Berkheij, 2005 ▸), being used as anti­fungal (Upadhayaya *et al.*, 2004 ▸), anti-bacterial, anti-malarial and anti-psychotic agents (Chaudhary *et al.*, 2006 ▸). An insight into advances on the anti­microbial activity of piperazine derivatives has been reported (Kharb *et al.*, 2012 ▸).

Piperazines are among the most important building blocks in today’s drug discovery and are found in biologically active compounds across a number of different therapeutic areas (Brockunier *et al.*, 2004 ▸; Bogatcheva *et al.*, 2006 ▸). A review of pharmacological and toxicological information for piperazine derivatives is given by Elliott (2011 ▸).

4-Nitro­phenyl­piperazinium chloride monohydrate has been used as an inter­mediate in the synthesis of anti­cancer drugs, transcriptase inhibitors and anti­fungal reagents and is also an important reagent for potassium channel openers, which show considerable biomolecular current-voltage rectification characteristics (Lu, 2007 ▸).

The inclusion behaviour of 4-sulfonato­calix[*n*]arenes (SCXn) (*n* = 4, 6, 8) with 1-(4-nitro­phen­yl)piperazine (NPP) has been investigated by UV spectroscopy and fluorescence spectroscopy at different pH values (Zhang *et al.*, 2014 ▸). The design, synthesis and biological profiling of aryl piperazine-based scaffolds for the management of androgen-sensitive prostatic disorders has been published (Gupta *et al.*, 2016 ▸). 4-Nitro­phenyl­piperazine was the starting material in the synthesis and biological evaluation of novel piperazine-containing hydrazone derivatives (Kaya *et al.*, 2016 ▸). Several previous investigations in this area are outlined in the *Database Survey* section.

In view of the importance of piperazines in general, and the use of 4-nitro­phenyl­piperazine in particular, the present paper reports the crystal structure studies of three salts of 4-nitro­phenyl­piperazine, *viz*., 4-nitro­phenyl­piperazinium salicylate (**1**), 4-nitro­phenyl­piperazinium 4-fluoro­benzoate trihydrate (**2**) and 4-nitro­phenyl­piperazinium 3,5-di­nitro­benzoate (**3**). 
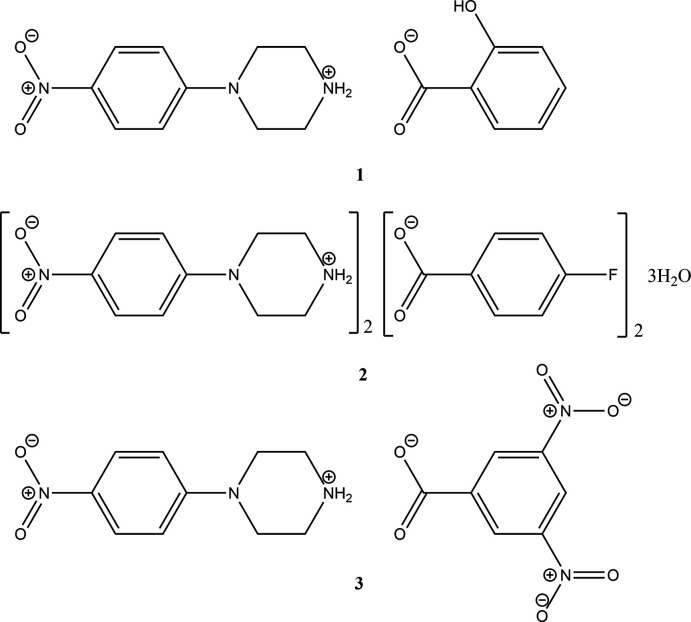


## 2. Structural commentary

**Figure 1:**
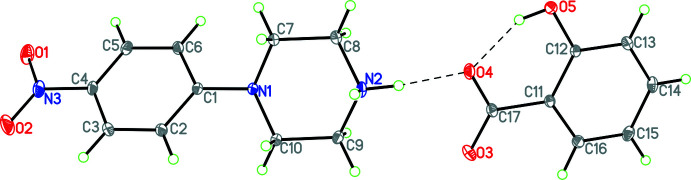
Diagram showing atom labelling and the arrangement of cation and anion in **1**. Hydrogen bonds are shown by dashed lines. Atomic displacement parameters are at the 30% probability level.

Compound **1**, (4-nitro­phenyl­piperazinium salicylate; C_10_H_14_N_3_O_2_·C_7_H_5_O_3_), crystallizes in the monoclinic space group *P2*_1_/*n w*ith four mol­ecules in the unit cell (Fig. 1 ▸). The structure contains a 4-phenyl­piperazinium cation linked to a salicylate anion by an N—H⋯O hydrogen bond [H⋯O = 1.792 (15) Å; N⋯O = 2.6957 (18) Å; N—H⋯O = 174.0 (18)°, Table 1 ▸]. In the salicylate anion, there is an intra­molecular hydrogen bond involving the phenol hydrogen and the carboxyl­ate group [ 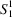 (6) in graph-set notation (Etter *et al.*, 1990 ▸): H⋯O = 1.733 (17) Å; O⋯O = 2.5221 (17) Å; O—H⋯O = 152 (2)°]. In the conformation of the cation and anion, the dihedral angles between the piperazine ring and the phenyl ring, the piperazine ring with the salicylate ring, the phenyl ring with the salicylate ring, the nitro group and the phenyl ring, and the salicylate ring and its carboxyl­ate group are 36.83 (6), 28.65 (6), 55.01 (5), 1.8 (3) and 7.0 (2)°, respectively. The first dihedral angle of 36.83 (6)° is indicative of the fact that the 4-nitro­phenyl ring occupies an equatorial position in the phenyl ring, with the nitro­gen lone pair occupying an axial position (see Fig. 1 ▸).

**Figure 2:**
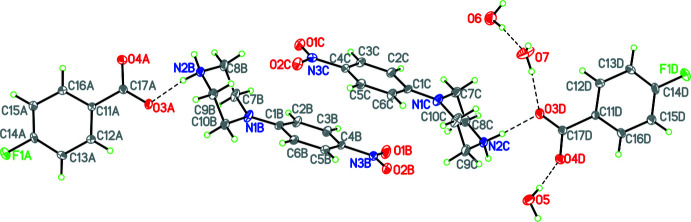
Diagram showing atom labelling indicating four rings *A*–*D*, the arrangement of cation and anion and the three water mol­ecules of solvation in **2**. Hydrogen bonds are shown by dashed lines. Atomic displacement parameters are at the 30% probability level.

Compound **2**, (4-nitro­phenyl­piperazinium 4-fluoro­benzoate; 2C_10_H_14_N_3_O_2_·2C_7_H_4_FO_2_·3H_2_O, crystallizes in the monoclinic space group *P2*_1_/*n* with four formula units in the unit cell. The structure consists of two 4-nitro­phenyl­piperazinium cations, two 4-fluoro­benzoate anions and three water solvate mol­ecules (see Fig. 2 ▸). Each cation is linked to a corresponding anion by a strong N—H⋯O hydrogen bond (Table 2 ▸). The water mol­ecules are also involved in hydrogen bonding, which will be discussed in further detail in section 3. As shown by Fig. 2 ▸, the structure has been divided into four rings, with rings *A* and *D* representing the two 4-fluoro­benzoate anions and rings *C* and *D* representing the two nitro­phenyl­piperazinium cations. Rings *A* and *B* are linked by a strong N—H⋯O hydrogen bond [H⋯O = 1.86 (2) Å; N⋯O = 2.739 (5) Å; N—H⋯O = 169 (4)°], as are rings *C* and *D* [H⋯O = 1.84 (2) Å; N⋯O = 2.733 (5)Å; N—H⋯O = 176 (4)°]. Additionally, ring *B*′s piperazine substituent forms a weak C—H⋯O inter­action with an oxygen atom in ring *C*′s terminal nitro group [H⋯O = 2.53 Å; C⋯O = 3.437 (6) Å; C—H⋯O = 155°]. Ring *C*′s piperazine substituent forms a similar inter­action with ring *B*′s terminal nitro group [H⋯O = 2.53 Å; C⋯O = 3.401 (6) Å; C—H⋯O = 149°]. In the conformation of rings *A*–*D*, the dihedral angles between the 4-nitro­phenyl rings in rings *B* and *C*, the 4-nitro­phenyl ring and nitro group in ring *B*, the 4-nitro­phenyl ring and nitro group in ring *C*, the piperazine ring and the 4-nitro­phenyl ring in ring *B*, the piperazine ring and 4-nitro­phenyl ring in ring *C*, the fluoro­benzene ring in ring *D* and the phenyl ring in ring *C*, and the fluoro­benzene ring in *A* and the phenyl ring in *C* are 11.4 (4), 1.1 (2), 0.2 (2), 141.72 (16), 145.17 (17), 101.47 (17) and 103.32 (17)°, respectively. The third and fourth angles listed indicate that the 4-nitro­phenyl ring occupies an axial position in both cations, which is relatively rare. In a previous paper containing eleven analogous structures, only one had this substitution pattern (Archana *et al.*, 2022 ▸).

**Figure 3:**
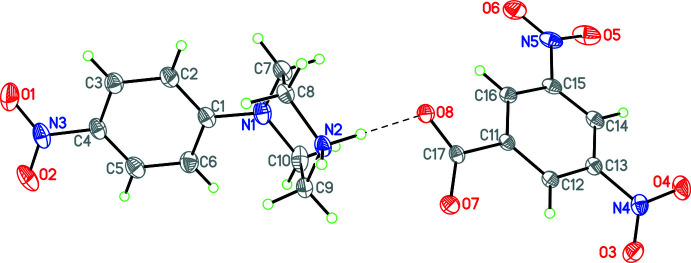
Diagram showing atom labelling and the arrangement of cation and anion in **3** (only major component shown). Hydrogen bonds are shown by dashed lines. Atomic displacement parameters are at the 30% probability level.

Compound **3**, (4-nitro­phenyl­piperazinium 3,5-di­nitro­benzoate; C_10_H_14_N_3_O_2_·C_7_H_4_N_2_O_6_), crystallizes in the monoclinic space group *C*2/*c* with eight formula units in the unit cell. The structure consists of a 4-nitro­phenyl­piperazinium cation and a 3,5-di­nitro­benzoate anion linked by a strong N—H⋯O hydrogen bond [H⋯O = 1.77 (2) Å; N⋯O = 2.705 (3) Å; N—H⋯O = 170.7 (17)°, Table 3 ▸] as shown in Fig. 3 ▸. The nitro­phenyl ring is disordered with occupancies of 0.806 (10)/0.194 (10). In the cation, the dihedral angles between the piperazine ring and the major component of the 4-nitro­phenyl ring, and the phenyl ring and its attached nitro group are 62.4 (1) and 10.1 (7)°, respectively. The former angle is indicative of the fact that the 4-nitro­phenyl ring occupies an axial position, as it also did in **2**. In the anion, the dihedral angle between the 3,5-di­nitro­benzoate phenyl ring and its carboxyl­ate substituent is 18.7 (1)°.

## 3. Supra­molecular features

**Figure 4:**
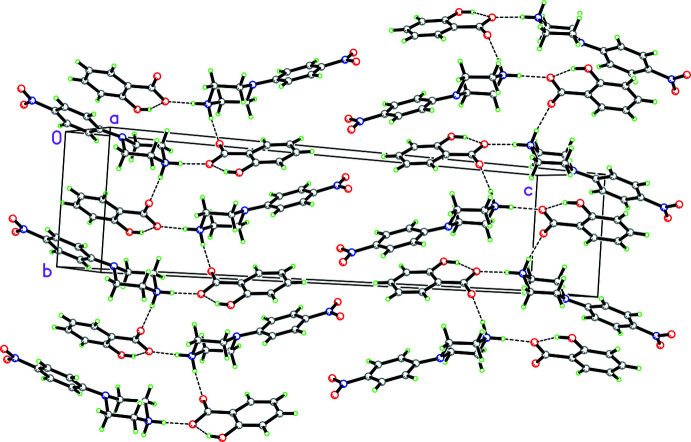
Packing diagram for **1** showing the zigzag chain of hydrogen bonds [graph-set notation 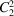 (6)] propagating in the *a*-axis direction.

**Figure 5:**
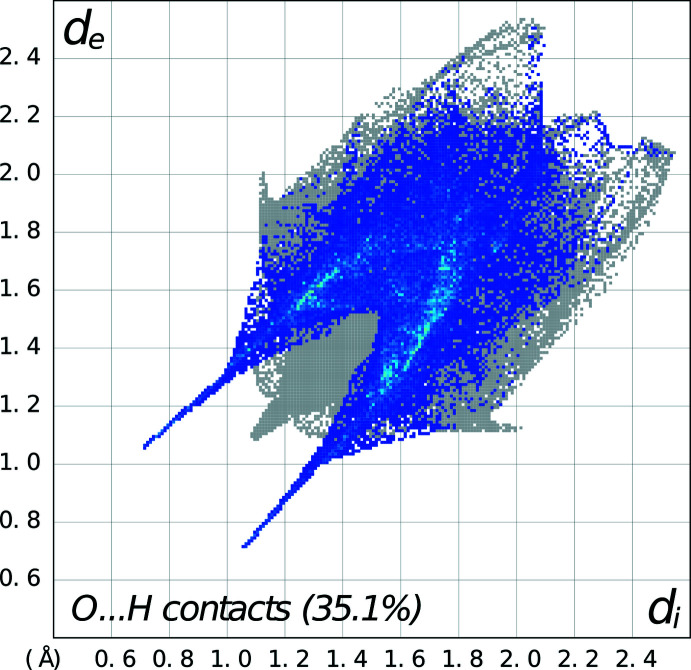
Fingerprint plot for **1** delineated into O⋯H/H⋯O contacts showing the prominent spikes indicating N—H⋯O hydrogen bonds.

In discussing the supra­molecular features of the three structures, the direct hydrogen bonding involving the linking of the 4-nitro­phenyl­piperazinium cations and organic acid anions is omitted since it has already been discussed in the previous section. For **1**, there is a zigzag chain of hydrogen bonds [graph-set notation 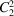 (6) (Etter *et al.*, 1990 ▸)], propagating in the *b*-axis direction involving the piperazinium cations and salicylate anions, as shown in Fig. 4 ▸. These are also illustrated in the Hirshfeld fingerprint plot (Spackman *et al.*, 2021 ▸), which shows the prominent spikes involving both types of N—H⋯O hydrogen bonding (see Fig. 5 ▸). In the packing of the piperazinium cation and the salicylate anion, the salicylate anion forms a π–π inter­action with the phenyl ring of a piperazinium cation [*Cg*2⋯*Cg*3^i^ distance = 3.9296 (2) Å; symmetry code: (i) 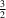 − *x*, *y* − 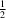 , 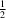 − *z*; slippage:1.505 Å; *Cg*2 and *Cg*3 are the centroids of the C1–C6 and C11–C16 rings, respectively]. Additionally, there is a C—H⋯π inter­action between a hydrogen atom in the piperazine ring and the phenyl ring within the salicyclate anion (H⋯*Cg* ^ii^ distance, 2.76 Å; C8—H8*B*⋯*Cg*2^ii^ angle of 156°, symmetry code: (ii) 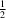 − *x*, 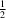 + *y*, 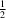 − *z*).

**Figure 6:**
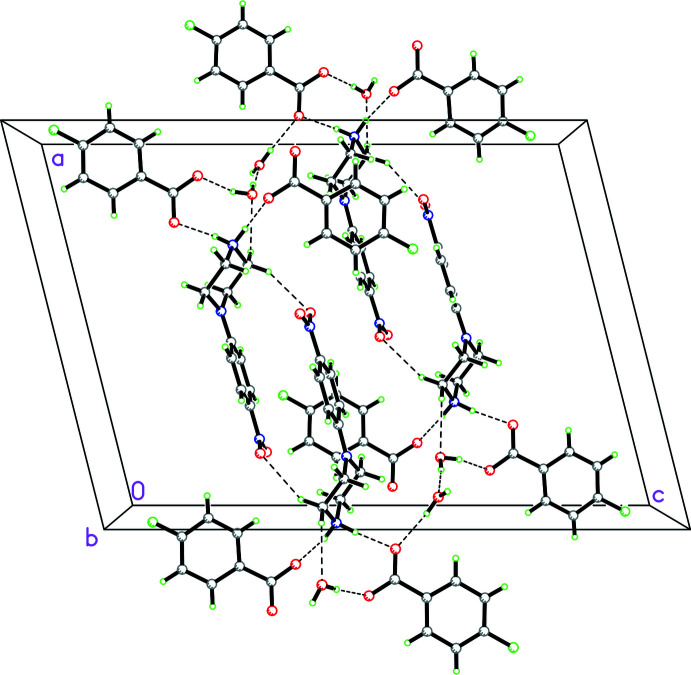
Packing diagram for **2** showing the complex three-dimensional array of hydrogen bonding involving both 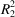 (20) motifs between rings *B* and *C* (middle left in diagram with ring *B* on right and ring *C* on left) and 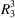 (9) motifs between rings *C* and *D* (ring *D* on upper left of diagram) as well as one water mol­ecule.

**Figure 7:**
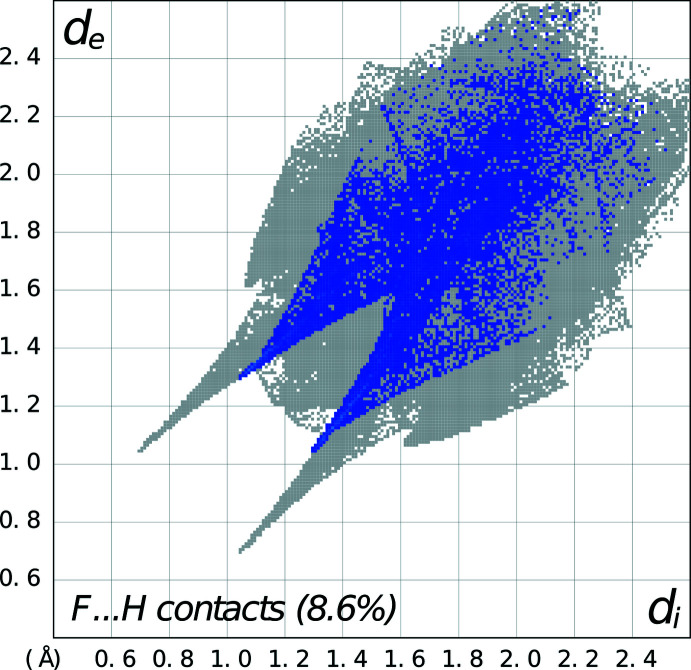
Fingerprint plot for **2** delineated into F⋯H/H⋯F contacts showing the prominent spikes as C—H⋯F inter­actions.

For **2**, there are two anions and two cations as well as three water mol­ecules of solvation in the asymmetric unit. This leads to a complex three-dimensional array of hydrogen bonding involving both 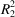 (20) motifs between rings *B* and *C*, and *R* ^3^_3_(9) motifs between rings *C* and *D* as well as one water mol­ecule, as seen in Fig. 6 ▸. There are also C—H⋯F inter­actions between adjacent fluoro­benzoate anions linking them into centrosymmetric dimers (symmetry code: 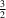 + *x*, 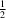 − *y*, − 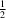 + *z*; see Table 2 ▸ for numerical details). These inter­actions are shown clearly as spikes in the fingerprint plots delineated into C—H⋯F and N—H⋯O inter­actions (Figs. 7 ▸ and 8 ▸, respectively). In the crystal, the phenyl ring in *B* forms an offset π–π inter­action with the phenyl ring in *C* [*Cg*2⋯*Cg*4 distance, 3.8568 (7) Å; slippage of 1.835 Å; perpendicular distance of 3.454 (2) Å; *Cg*2 and *Cg*4 are the centroids of the C1*B*–C6*B* and C1*C*–C6*C* rings, respectively]. Additionally, there is a C–H⋯π inter­action between a hydrogen atom on the phenyl ring in *C* and the phenyl ring in *D* [H6*C*⋯*Cg*6^i^, 2.91; C6—H6*C*⋯*Cg*6^i^ angle of 161°, symmetry code: (i) 1 − *x*, 1 − *y*, −*z*; *Cg*6 is the centroid of the C11*D*–C16*D* ring].

**Figure 9:**
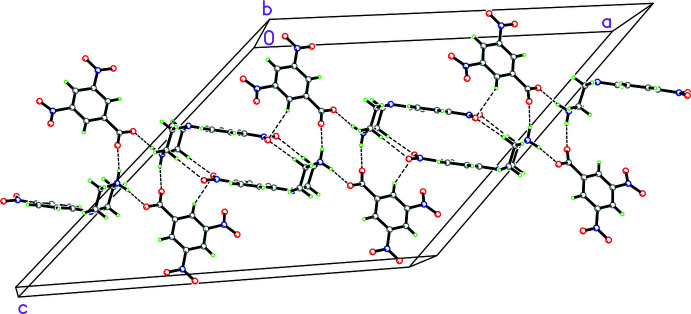
Packing diagram for **3** (only major component shown) showing the complex three-dimensional array of hydrogen bonding involving 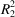 (8), 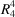 (12) and 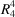 (20) rings propagating in the *a*-axis direction between the the 4-nitro­phenyl group of one cation with the piperazinium ring of an adjacent cation (symmetry code: 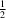 − *x*, 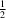 − *y*, 1 − *z*, involving O1^iii^ and O2^iii^; Table 4 ▸), two cations and two anions in adjacent asymmetric units (−*x*, −*y*, 1 − *z*, involving O7^i^), and two cations and two anions in adjacent asymmetric units (symmetry code: − 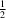 − *x*, 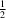 + *y*, 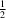 − *z*, involving O4^iv^) .

**Figure 10:**
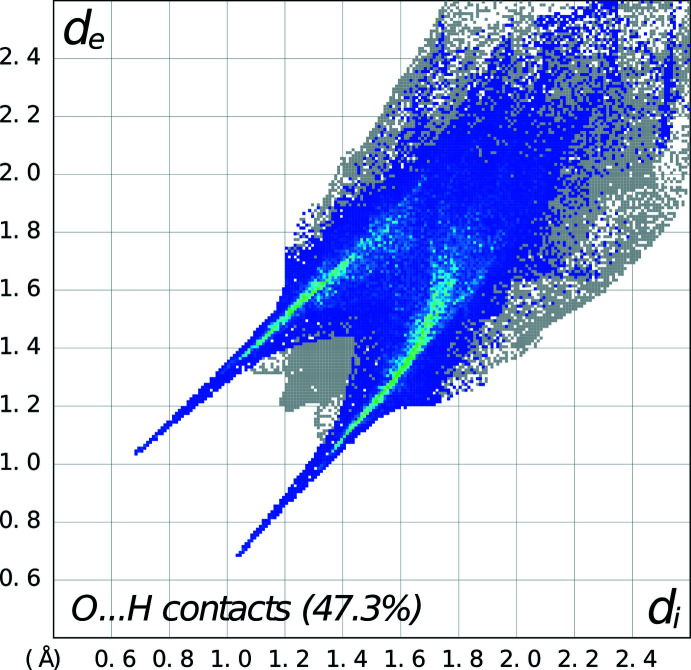
Fingerprint plot for **3** delineated into O⋯H/H⋯O contacts showing the prominent spikes as N—H⋯O inter­actions.

For **3**, the cation and the anion form a complex three-dimensional array of hydrogen bonding involving 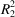 (8), 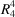 (12) and 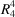 (20) rings propogating in the *a*-axis direction between the 4-nitro­phenyl group of one cation with the piperazinium ring of an adjacent cation (symmetry code: 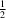 − *x*, 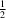 − *y*, 1 − *z*), two cations and two anions in adjacent asymmetric units (−*x*, −*y*, 1 − *z*), two cations and two anions in adjacent asymmetric units (symmetry code: − 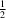 − *x*, 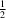 + *y*, 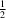 − *z*), respectively, as seen in Fig. 9 ▸ (Table 3 ▸). These show as sharp spikes in the fingerprint plot showing the N—H⋯O inter­actions (Fig. 10 ▸). Additionally, the nitro­benzene group within the piperazinium cation forms a π–π inter­action with the phenyl group of another piperazinium cation in an adjacent asymmetric unit [*Cg*2⋯*Cg*2^i^ distance, 4.4132 (9) Å; perpendicular distance: 3.5596 (9) Å; slippage of 2.609 Å, symmetry code: (i) 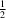 − *x*, 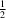 − *y*, 1 − *z*; *Cg*2 is the centroid of the C1–C6*D* ring].

## 4. Database survey

Related structures containing 1-phenyl­piperazine or the 1-phenyl­piperazinium cation include racemic perhydro­tri­phenyl­ene (PHTP), which has been shown to form a polar inclusion compound with 1-(4-nitro­phen­yl)piperazine (NPP) as a guest mol­ecule (CSD refcode NOVWOK; König *et al.*, 1997 ▸). The crystal structure of the simple salt 4-nitro­phen­ylpiperazinium chloride monohydrate has been reported (LIJNAU; Lu, 2007 ▸). The crystal structure of 4,6-di­meth­oxy­pyrimidin-2-amine-1-(4-nitro­phen­yl)piperazine (1:1) has been published (LUDMUU; Wang *et al.*, 2014 ▸) as well as the synthesis and crystal structure of a Schiff base, 5-methyl-2-{[4-(4-nitro­phen­yl)piperazin-1-yl]meth­yl}phenol (WUWBIC; Ayeni *et al.*, 2019 ▸).

NMR-based investigations of acyl-functionalized piperazines concerning their conformational behavior in solution has been studied and crystal structures of 1-(4-fluoro­benzo­yl)-4-(4-nitro­phen­yl)piperazine (BIQYIM), 1-(4-bromo­benzo­yl)-4-(4-nitro­phen­yl)piperazine (BIRHES), 1-(3-bromo­benzo­yl)-4-(4-nitro­phen­yl)piperazine (BIRHIW) and (piperazine-1,4-di­yl)bis­[(4-fluoro­phen­yl)methanone] (BIRGOB) have been reported (Wodtke *et al.*, 2018 ▸). We have recently reported the crystal structures of some salts of 4-meth­oxy­phenyl­piperazine (Kiran Kumar *et al.*, 2019 ▸) and also 2-meth­oxy­phenyl­piperazine (Harish Chinthal *et al.*, 2020 ▸). We have recently reported the crystal structures of some salts of piperazine derivatives (Archana *et al.*, 2021 ▸). Very recently, we have reported the crystal structures of six salts of 4-nitro­phenyl­piperazine (NEBVOJ; NEBVUP; NEBWAW; NEBWEA; NEBWIE; NEBWOK) and four salts of 1-phenyl­piperazine (Mahesha *et al.*, 2022*a* ▸, 2022*b* ▸). The syntheses and crystal structures of 4-(4-nitro­phen­yl)piperazin-1-ium benzoate monohydrate (BEFGIG) and 4-(4-nitro­phen­yl)piperazin-1-ium 2-carb­oxy-4,6-di­nitro­phenolate (BEFGOM) have been reported (Shankara Prasad *et al.*, 2022 ▸).

## 5. Synthesis and crystallization

**Figure 11:**
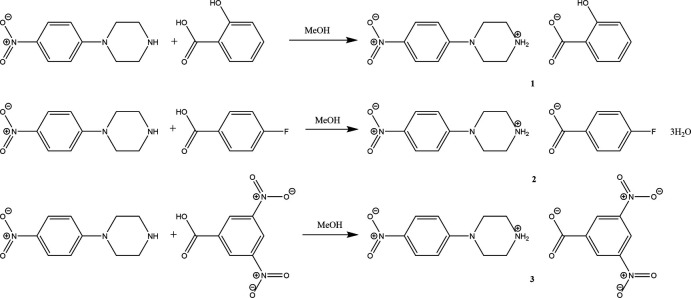
Reaction scheme for the synthesis of **1**, **2** and **3**.

For the synthesis of salts **1**–**3**, a solution of commercially available (from Sigma-Aldrich) 4-nitro­phenyl­piperazine (100 mg, 0.483 mol) in methanol (10 ml) was mixed with equimolar solutions of the appropriate acids in methanol (10 ml) and ethyl acetate (10 ml) *viz*., salicylic acid (67 mg) for **1**, 4-fluoro­benzoic acid (68 mg) for **2** and 3,5-di­nitro­benzoic acid (102 mg) for **3** (see Fig. 11 ▸ for reaction scheme). The corresponding solutions were stirred for 15 minutes at room temperature and allowed to stand at the same temperature. X-ray quality crystals were formed on slow evaporation (for **1** and **2**) for a week. For **3**, DMF (3 ml) was used for crystallization. The corresponding melting points were 453–458 K (**1**), 373–378 K (**2**) and 445–447 K (**3**).

## 6. Refinement

Crystal data, data collection and structure refinement details for the three structures are summarized in Table 4 ▸. In all structures, a riding model was used for the H atoms attached to C with *U*_iso_(H) = 1.2*U*_eq_(C) while the N–H and water O–H hydrogen atoms were refined isotropically. In **3** the nitro­phenyl group is disordered with occupancies of 0.806 (10)/0.194 (10) and constrained to have similar metrical parameters.

## Supplementary Material

Crystal structure: contains datablock(s) 1, 2, 3. DOI: 10.1107/S2056989023002517/vm2276sup1.cif

Structure factors: contains datablock(s) 1. DOI: 10.1107/S2056989023002517/vm22761sup2.hkl

Structure factors: contains datablock(s) 2. DOI: 10.1107/S2056989023002517/vm22762sup3.hkl

Structure factors: contains datablock(s) 3. DOI: 10.1107/S2056989023002517/vm22763sup4.hkl

Supporting information file. DOI: 10.1107/S2056989023002517/vm22761sup5.cml

Supporting information file. DOI: 10.1107/S2056989023002517/vm22762sup6.cml

Supporting information file. DOI: 10.1107/S2056989023002517/vm22763sup7.cml

CCDC references: 2248697, 2248696, 2248695

Additional supporting information:  crystallographic information; 3D view; checkCIF report

## Figures and Tables

**Figure 8:**
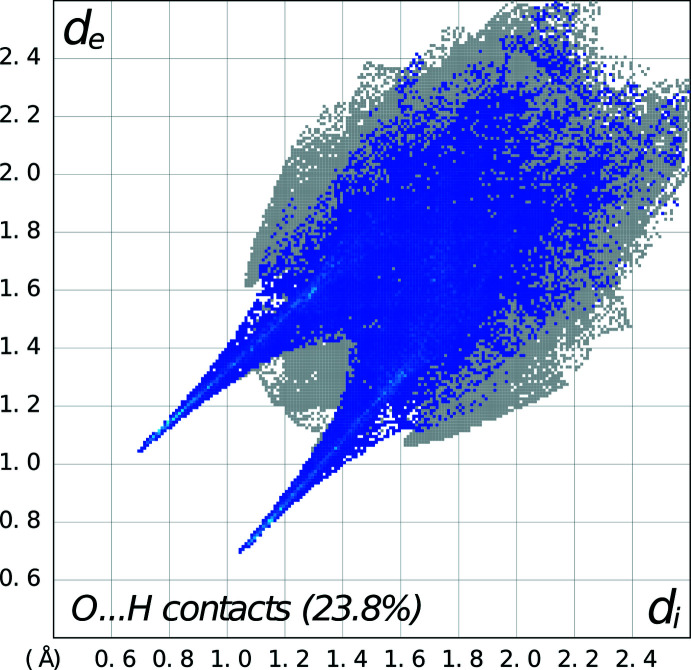
Fingerprint plot for **2** delineated into O⋯H/H⋯O contacts showing the prominent spikes as N—H⋯O inter­actions.

**Table 1:** Hydrogen-bond geometry (Å, °) for **1** [Chem scheme1]

*D*—H⋯*A*	*D*—H	H⋯*A*	*D*⋯*A*	*D*—H⋯*A*
C8—H8*A*⋯O5^i^	0.97	2.53	3.430 (2)	155
C9—H9*A*⋯O5^ii^	0.97	2.47	3.407 (2)	164
N2—H21⋯O3^iii^	0.92 (2)	1.88 (2)	2.784 (2)	170 (2)
N2—H22⋯O4	0.91 (2)	1.79 (2)	2.6957 (18)	174 (2)
O5—H5*O*⋯O4	0.86 (2)	1.73 (2)	2.5221 (17)	152 (2)

Symmetry codes: (i) 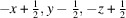 ; (ii) 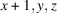 ; (iii) 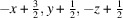 .

**Table 2:** Hydrogen-bond geometry (Å, °) for **2** [Chem scheme1]

*D*—H⋯*A*	*D*—H	H⋯*A*	*D*⋯*A*	*D*—H⋯*A*
N2*C*—H2*CA*⋯O3*D*	0.90 (2)	1.84 (2)	2.733 (5)	176 (4)
N2*C*—H2*CB*⋯O4*A* ^i^	0.87 (2)	1.87 (2)	2.726 (5)	166 (4)
C3*C*—H3*C*⋯O1*C* ^ii^	0.93	2.62	3.261 (7)	126
C9*C*—H9*CA*⋯O5	0.97	2.51	3.332 (6)	142
C9*C*—H9*CB*⋯O2*B*	0.97	2.53	3.401 (6)	149
C15*D*—H15*D*⋯F1*A* ^iii^	0.93	2.52	3.369 (5)	152
N2*B*—H2*BA*⋯O4*D* ^iv^	0.88 (2)	1.92 (2)	2.764 (5)	163 (4)
N2*B*—H2*BB*⋯O3*A*	0.89 (2)	1.86 (2)	2.739 (5)	169 (4)
C3*B*—H3*B*⋯F1*D* ^v^	0.93	2.63	3.436 (6)	145
C8*B*—H8*BA*⋯O1*C*	0.97	2.53	3.437 (6)	155
C8*B*—H8*BB*⋯O6^ii^	0.97	2.49	3.182 (6)	129
C15*A*—H15*A*⋯F1*D* ^vi^	0.93	2.48	3.310 (5)	149
O5—H5*A*⋯O3*A* ^vii^	0.84 (2)	1.96 (2)	2.794 (4)	172 (5)
O5—H5*D*⋯O4*D*	0.83 (2)	1.95 (2)	2.778 (5)	175 (5)
O6—H6*A*⋯O4*A* ^ii^	0.84 (2)	2.04 (4)	2.795 (6)	149 (7)
O6—H6*D*⋯O7	0.84 (2)	1.91 (2)	2.744 (7)	174 (8)
O7—H7*A*⋯O3*D*	0.83 (2)	1.99 (3)	2.794 (5)	163 (7)
O7—H7*B*⋯O5^viii^	0.83 (2)	1.93 (2)	2.759 (6)	172 (7)

Symmetry codes: (i) 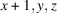 ; (ii) 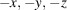 ; (iii) 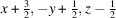 ; (iv) 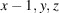 ; (v) 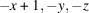 ; (vi) 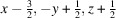 ; (vii) 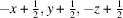 ; (viii) 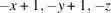 .

**Table 3:** Hydrogen-bond geometry (Å, °) for **3** [Chem scheme1]

*D*—H⋯*A*	*D*—H	H⋯*A*	*D*⋯*A*	*D*—H⋯*A*
N2—H21⋯O8	0.95 (2)	1.77 (2)	2.705 (3)	170.7 (17)
N2—H22⋯O7^i^	0.92 (2)	1.81 (2)	2.715 (3)	166.4 (18)
C8—H8*A*⋯O4^ii^	0.97	2.54	3.245 (3)	130
C8—H8*B*⋯O2^iii^	0.97	2.40	3.354 (4)	168
C8—H8*B*⋯O2*A* ^iii^	0.97	2.39	3.353 (16)	172
C9—H9*A*⋯O1^iii^	0.97	2.54	3.497 (5)	171
C9—H9*A*⋯O1*A* ^iii^	0.97	2.47	3.378 (14)	155
C10—H10*B*⋯O4^iv^	0.97	2.60	3.425 (3)	143
C12—H12⋯O2^v^	0.93	2.53	3.231 (4)	133

Symmetry codes: (i) 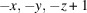 ; (ii) 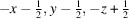 ; (iii) 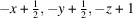 ; (iv) 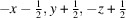 ; (v) 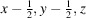 .

**Table 4:** Experimental details

	**1**	**2**	**3**
Crystal data			
Chemical formula	C_10_H_14_N_3_O_2_^+^·C_7_H_5_O_3_^−^	2C_10_H_14_N_3_O_2_^+^·2C_7_H_4_FO_2_^−^·3H_2_O	C_10_H_14_N_3_O_2_^+^·C_7_H_3_N_2_O_6_^−^
*M*_r_	345.35	748.73	419.35
Crystal system, space group	Monoclinic, *P*2_1_/*n*	Monoclinic, *P*2_1_/*n*	Monoclinic, *C*2/*c*
Temperature (K)	293	293	293
*a*, *b*, *c* (Å)	7.0018 (3), 7.3938 (3), 31.531 (1)	16.882 (2), 9.719 (1), 23.445 (4)	27.953 (6), 8.1422 (6), 24.657 (5)
β (°)	90.132 (4)	104.17 (1)	136.55 (4)
*V* (Å^3^)	1632.35 (11)	3729.7 (9)	3859 (2)
*Z*	4	4	8
Radiation type	Mo *K*α	Mo *K*α	Mo *K*α
μ (mm^−1^)	0.11	0.11	0.12
Crystal size (mm)	0.48 × 0.40 × 0.40	0.32 × 0.16 × 0.14	0.48 × 0.48 × 0.40

Data collection			
Diffractometer	Oxford Diffraction Xcalibur with Sapphire CCD	Oxford Diffraction Xcalibur with Sapphire CCD	Oxford Diffraction Xcalibur with Sapphire CCD
Absorption correction	Multi-scan (*CrysAlis RED*; Oxford Diffraction, 2009 ▸)	Multi-scan (*CrysAlis RED*; Oxford Diffraction, 2009 ▸)	Multi-scan (*CrysAlis RED*; Oxford Diffraction, 2009 ▸)
*T*_min_, *T*_max_	0.886, 1.000	0.887, 1.000	0.621, 1.000
No. of measured, independent and observed [*I* > 2σ(*I*)] reflections	6955, 3506, 2606	14552, 6755, 2429	8107, 4119, 2838
*R*_int_	0.016	0.063	0.015
(sin θ/λ)_max_ (Å^−1^)	0.655	0.602	0.655

Refinement			
*R*[*F* ^2^ > 2σ(*F* ^2^)], *wR*(*F* ^2^), *S*	0.049, 0.119, 1.04	0.089, 0.154, 1.06	0.044, 0.119, 1.03
No. of reflections	3506	6755	4119
No. of parameters	235	508	337
No. of restraints	3	10	262
H-atom treatment	H atoms treated by a mixture of independent and constrained refinement	H atoms treated by a mixture of independent and constrained refinement	H atoms treated by a mixture of independent and constrained refinement
Δρ_max_, Δρ_min_ (e Å^−3^)	0.22, −0.21	0.19, −0.18	0.24, −0.24

Computer programs: *CrysAlis CCD* and *CrysAlis RED* (Oxford Diffraction, 2009 ▸), *SHELXT* (Sheldrick, 2015*a* ▸), *SHELXL2018/3* (Sheldrick, 2015*b* ▸) and *SHELXTL* (Sheldrick, 2008 ▸).

## supplementary crystallographic information

### 4-(4-Nitrophenyl)piperazin-1-ium 2-hydroxybenzoate (1) Crystal data

**Table:**

C_10_H_14_N_3_O_2_^+^·C_7_H_5_O_3_^−^	*F*(000) = 728
*M**_r_* = 345.35	*D*_x_ = 1.405 Mg m^−^^3^
Monoclinic, *P*2_1_/*n*	Mo *K*α radiation, λ = 0.71073 Å
*a* = 7.0018 (3) Å	Cell parameters from 3505 reflections
*b* = 7.3938 (3) Å	θ = 2.6–27.7°
*c* = 31.531 (1) Å	µ = 0.11 mm^−^^1^
β = 90.132 (4)°	*T* = 293 K
*V* = 1632.35 (11) Å^3^	Prism, yellow
*Z* = 4	0.48 × 0.40 × 0.40 mm

### 4-(4-Nitrophenyl)piperazin-1-ium 2-hydroxybenzoate (1) Data collection

**Table:**

Oxford Diffraction Xcalibur with Sapphire CCD diffractometer	2606 reflections with *I* > 2σ(*I*)
Radiation source: Enhance (Mo) X-ray Source	*R*_int_ = 0.016
Rotation method data acquisition using ω scans.	θ_max_ = 27.8°, θ_min_ = 2.6°
Absorption correction: multi-scan (CrysAlis RED; Oxford Diffraction, 2009)	*h* = −8→7
*T*_min_ = 0.886, *T*_max_ = 1.000	*k* = −5→9
6955 measured reflections	*l* = −30→41
3506 independent reflections	

### 4-(4-Nitrophenyl)piperazin-1-ium 2-hydroxybenzoate (1) Refinement

**Table:**

Refinement on *F*^2^	Primary atom site location: dual
Least-squares matrix: full	Secondary atom site location: difference Fourier map
*R*[*F*^2^ > 2σ(*F*^2^)] = 0.049	Hydrogen site location: mixed
*wR*(*F*^2^) = 0.119	H atoms treated by a mixture of independent and constrained refinement
*S* = 1.04	*w* = 1/[σ^2^(*F*_o_^2^) + (0.0464*P*)^2^ + 0.6706*P*] where *P* = (*F*_o_^2^ + 2*F*_c_^2^)/3
3506 reflections	(Δ/σ)_max_ < 0.001
235 parameters	Δρ_max_ = 0.22 e Å^−^^3^
3 restraints	Δρ_min_ = −0.21 e Å^−^^3^

### 4-(4-Nitrophenyl)piperazin-1-ium 2-hydroxybenzoate (1) Special details

**Table:**

Geometry. All esds (except the esd in the dihedral angle between two l.s. planes) are estimated using the full covariance matrix. The cell esds are taken into account individually in the estimation of esds in distances, angles and torsion angles; correlations between esds in cell parameters are only used when they are defined by crystal symmetry. An approximate (isotropic) treatment of cell esds is used for estimating esds involving l.s. planes.

### 4-(4-Nitrophenyl)piperazin-1-ium 2-hydroxybenzoate (1) Fractional atomic coordinates and isotropic or equivalent isotropic displacement parameters (Å^2^)

**Table:**

	*x*	*y*	*z*	*U*_iso_*/*U*_eq_	
C1	0.9480 (2)	0.4305 (2)	0.40335 (5)	0.0322 (4)	
C2	1.1448 (3)	0.4516 (3)	0.40783 (6)	0.0420 (4)	
H2	1.214403	0.506875	0.386354	0.050*	
C3	1.2372 (3)	0.3912 (3)	0.44378 (6)	0.0481 (5)	
H3	1.368446	0.406601	0.446657	0.058*	
C4	1.1344 (3)	0.3082 (3)	0.47539 (5)	0.0410 (4)	
C5	0.9401 (3)	0.2866 (3)	0.47224 (6)	0.0435 (4)	
H5	0.871988	0.231148	0.493930	0.052*	
C6	0.8476 (3)	0.3485 (3)	0.43645 (6)	0.0416 (4)	
H6	0.715817	0.335531	0.434273	0.050*	
C7	0.6915 (2)	0.6105 (3)	0.37246 (5)	0.0376 (4)	
H7A	0.742612	0.729932	0.378242	0.045*	
H7B	0.616032	0.573640	0.396727	0.045*	
C8	0.5651 (2)	0.6186 (3)	0.33363 (5)	0.0380 (4)	
H8A	0.499976	0.503859	0.330013	0.046*	
H8B	0.469135	0.711902	0.337361	0.046*	
C9	0.8353 (3)	0.5226 (3)	0.29013 (5)	0.0423 (4)	
H9A	0.909732	0.550803	0.265077	0.051*	
H9B	0.780236	0.403244	0.286470	0.051*	
C10	0.9632 (2)	0.5244 (3)	0.32892 (5)	0.0371 (4)	
H10A	1.064043	0.435557	0.325689	0.045*	
H10B	1.021744	0.642570	0.332035	0.045*	
C11	0.3106 (2)	0.5498 (2)	0.16617 (5)	0.0327 (4)	
C12	0.1281 (2)	0.6228 (2)	0.17173 (5)	0.0322 (4)	
C13	−0.0084 (3)	0.6071 (3)	0.13974 (6)	0.0407 (4)	
H13	−0.130219	0.654106	0.143688	0.049*	
C14	0.0370 (3)	0.5219 (3)	0.10231 (6)	0.0462 (5)	
H14	−0.055100	0.510333	0.081179	0.055*	
C15	0.2181 (3)	0.4534 (3)	0.09584 (6)	0.0500 (5)	
H15	0.248883	0.397864	0.070291	0.060*	
C16	0.3527 (3)	0.4681 (3)	0.12755 (6)	0.0434 (5)	
H16	0.474660	0.422323	0.123046	0.052*	
C17	0.4577 (2)	0.5577 (2)	0.20097 (6)	0.0383 (4)	
N1	0.84988 (19)	0.4826 (2)	0.36656 (4)	0.0346 (3)	
N2	0.6802 (2)	0.6586 (2)	0.29534 (5)	0.0402 (4)	
H21	0.735 (3)	0.771 (2)	0.2977 (6)	0.048*	
H22	0.597 (2)	0.659 (3)	0.2733 (5)	0.048*	
N3	1.2348 (3)	0.2403 (3)	0.51271 (6)	0.0578 (5)	
O1	1.1425 (3)	0.1642 (3)	0.54024 (5)	0.0781 (5)	
O2	1.4076 (3)	0.2658 (3)	0.51533 (6)	0.0970 (7)	
O3	0.61169 (19)	0.4778 (2)	0.19676 (5)	0.0570 (4)	
O4	0.41348 (19)	0.6511 (2)	0.23358 (4)	0.0500 (4)	
O5	0.07825 (18)	0.7116 (2)	0.20780 (4)	0.0468 (3)	
H5O	0.178 (2)	0.710 (3)	0.2234 (6)	0.056*	

### 4-(4-Nitrophenyl)piperazin-1-ium 2-hydroxybenzoate (1) Atomic displacement parameters (Å^2^)

**Table:**

	*U*^11^	*U*^22^	*U*^33^	*U*^12^	*U*^13^	*U*^23^
C1	0.0357 (9)	0.0313 (9)	0.0295 (8)	0.0005 (7)	−0.0032 (7)	−0.0013 (7)
C2	0.0369 (9)	0.0525 (11)	0.0367 (9)	0.0005 (8)	−0.0012 (7)	0.0080 (8)
C3	0.0357 (9)	0.0647 (13)	0.0439 (11)	0.0055 (9)	−0.0085 (8)	0.0037 (10)
C4	0.0503 (11)	0.0412 (10)	0.0315 (9)	0.0072 (9)	−0.0107 (8)	0.0010 (8)
C5	0.0532 (11)	0.0445 (11)	0.0329 (9)	−0.0067 (9)	−0.0016 (8)	0.0052 (8)
C6	0.0366 (9)	0.0507 (11)	0.0374 (9)	−0.0081 (8)	−0.0053 (7)	0.0048 (8)
C7	0.0338 (9)	0.0470 (11)	0.0320 (9)	0.0031 (8)	−0.0028 (7)	−0.0005 (8)
C8	0.0354 (9)	0.0405 (10)	0.0382 (9)	−0.0016 (8)	−0.0072 (7)	0.0009 (8)
C9	0.0453 (10)	0.0538 (12)	0.0279 (9)	−0.0024 (9)	−0.0022 (7)	−0.0002 (8)
C10	0.0359 (9)	0.0453 (10)	0.0301 (9)	0.0003 (8)	−0.0014 (7)	0.0010 (8)
C11	0.0349 (9)	0.0287 (8)	0.0344 (9)	0.0014 (7)	−0.0043 (7)	0.0036 (7)
C12	0.0341 (8)	0.0309 (9)	0.0317 (8)	−0.0008 (7)	−0.0033 (7)	0.0027 (7)
C13	0.0348 (9)	0.0412 (10)	0.0462 (10)	0.0003 (8)	−0.0078 (8)	0.0045 (8)
C14	0.0573 (12)	0.0411 (11)	0.0400 (10)	−0.0038 (9)	−0.0191 (9)	0.0009 (8)
C15	0.0727 (14)	0.0436 (11)	0.0337 (10)	0.0077 (10)	−0.0050 (9)	−0.0050 (8)
C16	0.0475 (11)	0.0419 (10)	0.0409 (10)	0.0124 (9)	0.0005 (8)	0.0005 (8)
C17	0.0369 (9)	0.0351 (9)	0.0429 (10)	−0.0007 (8)	−0.0076 (8)	0.0074 (8)
N1	0.0338 (7)	0.0434 (8)	0.0267 (7)	0.0016 (6)	−0.0025 (6)	0.0036 (6)
N2	0.0442 (9)	0.0431 (9)	0.0333 (8)	−0.0078 (7)	−0.0150 (6)	0.0049 (7)
N3	0.0719 (13)	0.0590 (11)	0.0423 (10)	0.0081 (10)	−0.0188 (9)	0.0060 (9)
O1	0.0997 (13)	0.0862 (13)	0.0481 (9)	−0.0073 (11)	−0.0204 (9)	0.0270 (9)
O2	0.0658 (11)	0.1458 (19)	0.0794 (13)	0.0075 (12)	−0.0338 (9)	0.0346 (12)
O3	0.0401 (8)	0.0599 (9)	0.0710 (10)	0.0141 (7)	−0.0141 (7)	0.0035 (8)
O4	0.0517 (8)	0.0608 (9)	0.0375 (7)	0.0066 (7)	−0.0175 (6)	−0.0052 (7)
O5	0.0427 (7)	0.0608 (9)	0.0368 (7)	0.0106 (7)	−0.0033 (5)	−0.0085 (6)

### 4-(4-Nitrophenyl)piperazin-1-ium 2-hydroxybenzoate (1) Geometric parameters (Å, º)

**Table:**

C1—C2	1.394 (2)	C10—N1	1.462 (2)
C1—C6	1.398 (2)	C10—H10A	0.9700
C1—N1	1.401 (2)	C10—H10B	0.9700
C2—C3	1.378 (2)	C11—C16	1.391 (2)
C2—H2	0.9300	C11—C12	1.399 (2)
C3—C4	1.375 (3)	C11—C17	1.504 (2)
C3—H3	0.9300	C12—O5	1.359 (2)
C4—C5	1.373 (3)	C12—C13	1.393 (2)
C4—N3	1.458 (2)	C13—C14	1.375 (3)
C5—C6	1.378 (2)	C13—H13	0.9300
C5—H5	0.9300	C14—C15	1.381 (3)
C6—H6	0.9300	C14—H14	0.9300
C7—N1	1.470 (2)	C15—C16	1.376 (3)
C7—C8	1.510 (2)	C15—H15	0.9300
C7—H7A	0.9700	C16—H16	0.9300
C7—H7B	0.9700	C17—O3	1.237 (2)
C8—N2	1.483 (2)	C17—O4	1.277 (2)
C8—H8A	0.9700	N2—H21	0.918 (15)
C8—H8B	0.9700	N2—H22	0.907 (15)
C9—N2	1.489 (2)	N3—O1	1.221 (2)
C9—C10	1.514 (2)	N3—O2	1.227 (3)
C9—H9A	0.9700	O5—H5O	0.855 (15)
C9—H9B	0.9700		
			
C2—C1—C6	118.13 (15)	C9—C10—H10A	109.8
C2—C1—N1	122.42 (15)	N1—C10—H10B	109.8
C6—C1—N1	119.41 (15)	C9—C10—H10B	109.8
C3—C2—C1	120.64 (17)	H10A—C10—H10B	108.2
C3—C2—H2	119.7	C16—C11—C12	118.20 (15)
C1—C2—H2	119.7	C16—C11—C17	120.64 (16)
C4—C3—C2	119.66 (17)	C12—C11—C17	121.15 (15)
C4—C3—H3	120.2	O5—C12—C13	117.97 (15)
C2—C3—H3	120.2	O5—C12—C11	121.87 (14)
C5—C4—C3	121.31 (16)	C13—C12—C11	120.15 (16)
C5—C4—N3	119.60 (18)	C14—C13—C12	120.03 (17)
C3—C4—N3	119.09 (18)	C14—C13—H13	120.0
C4—C5—C6	118.97 (18)	C12—C13—H13	120.0
C4—C5—H5	120.5	C13—C14—C15	120.56 (17)
C6—C5—H5	120.5	C13—C14—H14	119.7
C5—C6—C1	121.28 (17)	C15—C14—H14	119.7
C5—C6—H6	119.4	C16—C15—C14	119.40 (18)
C1—C6—H6	119.4	C16—C15—H15	120.3
N1—C7—C8	111.35 (14)	C14—C15—H15	120.3
N1—C7—H7A	109.4	C15—C16—C11	121.61 (17)
C8—C7—H7A	109.4	C15—C16—H16	119.2
N1—C7—H7B	109.4	C11—C16—H16	119.2
C8—C7—H7B	109.4	O3—C17—O4	123.89 (16)
H7A—C7—H7B	108.0	O3—C17—C11	119.89 (17)
N2—C8—C7	110.44 (14)	O4—C17—C11	116.22 (15)
N2—C8—H8A	109.6	C1—N1—C10	117.67 (13)
C7—C8—H8A	109.6	C1—N1—C7	116.15 (13)
N2—C8—H8B	109.6	C10—N1—C7	112.18 (13)
C7—C8—H8B	109.6	C8—N2—C9	110.67 (14)
H8A—C8—H8B	108.1	C8—N2—H21	109.9 (13)
N2—C9—C10	109.57 (14)	C9—N2—H21	108.5 (12)
N2—C9—H9A	109.8	C8—N2—H22	105.9 (12)
C10—C9—H9A	109.8	C9—N2—H22	112.8 (13)
N2—C9—H9B	109.8	H21—N2—H22	109.1 (18)
C10—C9—H9B	109.8	O1—N3—O2	123.11 (18)
H9A—C9—H9B	108.2	O1—N3—C4	118.53 (19)
N1—C10—C9	109.44 (14)	O2—N3—C4	118.3 (2)
N1—C10—H10A	109.8	C12—O5—H5O	105.3 (15)
			
C6—C1—C2—C3	0.7 (3)	C12—C11—C16—C15	−2.0 (3)
N1—C1—C2—C3	−177.07 (17)	C17—C11—C16—C15	177.55 (18)
C1—C2—C3—C4	0.5 (3)	C16—C11—C17—O3	−6.2 (3)
C2—C3—C4—C5	−1.1 (3)	C12—C11—C17—O3	173.34 (17)
C2—C3—C4—N3	178.25 (18)	C16—C11—C17—O4	173.08 (17)
C3—C4—C5—C6	0.5 (3)	C12—C11—C17—O4	−7.4 (2)
N3—C4—C5—C6	−178.85 (17)	C2—C1—N1—C10	12.9 (2)
C4—C5—C6—C1	0.7 (3)	C6—C1—N1—C10	−164.78 (16)
C2—C1—C6—C5	−1.3 (3)	C2—C1—N1—C7	−124.13 (18)
N1—C1—C6—C5	176.53 (17)	C6—C1—N1—C7	58.1 (2)
N1—C7—C8—N2	−54.0 (2)	C9—C10—N1—C1	163.32 (15)
N2—C9—C10—N1	59.2 (2)	C9—C10—N1—C7	−58.00 (19)
C16—C11—C12—O5	−177.19 (16)	C8—C7—N1—C1	−164.92 (15)
C17—C11—C12—O5	3.3 (3)	C8—C7—N1—C10	55.73 (19)
C16—C11—C12—C13	2.3 (3)	C7—C8—N2—C9	56.27 (19)
C17—C11—C12—C13	−177.21 (16)	C10—C9—N2—C8	−59.12 (19)
O5—C12—C13—C14	178.56 (17)	C5—C4—N3—O1	0.6 (3)
C11—C12—C13—C14	−1.0 (3)	C3—C4—N3—O1	−178.8 (2)
C12—C13—C14—C15	−0.8 (3)	C5—C4—N3—O2	−177.9 (2)
C13—C14—C15—C16	1.2 (3)	C3—C4—N3—O2	2.7 (3)
C14—C15—C16—C11	0.3 (3)		

### 4-(4-Nitrophenyl)piperazin-1-ium 2-hydroxybenzoate (1) Hydrogen-bond geometry (Å, º)

**Table:**

*D*—H···*A*	*D*—H	H···*A*	*D*···*A*	*D*—H···*A*
C8—H8*A*···O5^i^	0.97	2.53	3.430 (2)	155
C9—H9*A*···O5^ii^	0.97	2.47	3.407 (2)	164
N2—H21···O3^iii^	0.92 (2)	1.88 (2)	2.784 (2)	170 (2)
N2—H22···O4	0.91 (2)	1.79 (2)	2.6957 (18)	174 (2)
O5—H5*O*···O4	0.86 (2)	1.73 (2)	2.5221 (17)	152 (2)

Symmetry codes: (i) −*x*+1/2, *y*−1/2, −*z*+1/2; (ii) *x*+1, *y*, *z*; (iii) −*x*+3/2, *y*+1/2, −*z*+1/2.

### Bis[4-(4-nitrophenyl)piperazin-1-ium] bis(4-fluorobenzoate) trihydrate (2) Crystal data

**Table:**

2C_10_H_14_N_3_O_2_^+^·2C_7_H_4_FO_2_^−^·3H_2_O	*F*(000) = 1576
*M**_r_* = 748.73	*D*_x_ = 1.333 Mg m^−^^3^
Monoclinic, *P*2_1_/*n*	Mo *K*α radiation, λ = 0.71073 Å
*a* = 16.882 (2) Å	Cell parameters from 3000 reflections
*b* = 9.719 (1) Å	θ = 2.7–27.9°
*c* = 23.445 (4) Å	µ = 0.11 mm^−^^1^
β = 104.17 (1)°	*T* = 293 K
*V* = 3729.7 (9) Å^3^	Prism, yellow
*Z* = 4	0.32 × 0.16 × 0.14 mm

### Bis[4-(4-nitrophenyl)piperazin-1-ium] bis(4-fluorobenzoate) trihydrate (2) Data collection

**Table:**

Oxford Diffraction Xcalibur with Sapphire CCD diffractometer	2429 reflections with *I* > 2σ(*I*)
Radiation source: Enhance (Mo) X-ray Source	*R*_int_ = 0.063
Rotation method data acquisition using ω scans.	θ_max_ = 25.4°, θ_min_ = 2.7°
Absorption correction: multi-scan (CrysAlis RED; Oxford Diffraction, 2009)	*h* = −18→20
*T*_min_ = 0.887, *T*_max_ = 1.000	*k* = −10→11
14552 measured reflections	*l* = −27→28
6755 independent reflections	

### Bis[4-(4-nitrophenyl)piperazin-1-ium] bis(4-fluorobenzoate) trihydrate (2) Refinement

**Table:**

Refinement on *F*^2^	Primary atom site location: dual
Least-squares matrix: full	Secondary atom site location: difference Fourier map
*R*[*F*^2^ > 2σ(*F*^2^)] = 0.089	Hydrogen site location: mixed
*wR*(*F*^2^) = 0.154	H atoms treated by a mixture of independent and constrained refinement
*S* = 1.06	*w* = 1/[σ^2^(*F*_o_^2^) + (0.0366*P*)^2^ + 1.3296*P*] where *P* = (*F*_o_^2^ + 2*F*_c_^2^)/3
6755 reflections	(Δ/σ)_max_ < 0.001
508 parameters	Δρ_max_ = 0.19 e Å^−^^3^
10 restraints	Δρ_min_ = −0.18 e Å^−^^3^

### Bis[4-(4-nitrophenyl)piperazin-1-ium] bis(4-fluorobenzoate) trihydrate (2) Special details

**Table:**

Geometry. All esds (except the esd in the dihedral angle between two l.s. planes) are estimated using the full covariance matrix. The cell esds are taken into account individually in the estimation of esds in distances, angles and torsion angles; correlations between esds in cell parameters are only used when they are defined by crystal symmetry. An approximate (isotropic) treatment of cell esds is used for estimating esds involving l.s. planes.

### Bis[4-(4-nitrophenyl)piperazin-1-ium] bis(4-fluorobenzoate) trihydrate (2) Fractional atomic coordinates and isotropic or equivalent isotropic displacement parameters (Å^2^)

**Table:**

	*x*	*y*	*z*	*U*_iso_*/*U*_eq_	
O1C	−0.0312 (2)	0.0993 (6)	0.05767 (19)	0.1313 (19)	
O2C	−0.0276 (2)	0.3166 (5)	0.07469 (19)	0.1200 (17)	
N1C	0.3330 (2)	0.2257 (4)	0.05682 (18)	0.0771 (13)	
N2C	0.5029 (2)	0.2032 (6)	0.10503 (19)	0.0755 (13)	
H2CA	0.525 (3)	0.228 (5)	0.0755 (15)	0.091*	
H2CB	0.5443 (19)	0.181 (4)	0.1336 (15)	0.091*	
N3C	0.0042 (3)	0.2079 (7)	0.0642 (2)	0.0969 (18)	
C1C	0.2521 (3)	0.2222 (5)	0.05718 (18)	0.0548 (12)	
C2C	0.2079 (3)	0.1001 (5)	0.0481 (2)	0.0711 (15)	
H2C	0.233224	0.019457	0.040707	0.085*	
C3C	0.1267 (3)	0.0971 (6)	0.0501 (2)	0.0772 (16)	
H3C	0.097724	0.014854	0.044109	0.093*	
C4C	0.0892 (3)	0.2159 (7)	0.0607 (2)	0.0682 (15)	
C5C	0.1305 (3)	0.3366 (6)	0.0683 (2)	0.0733 (15)	
H5C	0.104210	0.417170	0.074714	0.088*	
C6C	0.2101 (3)	0.3398 (5)	0.0666 (2)	0.0683 (14)	
H6C	0.237594	0.423523	0.071920	0.082*	
C7C	0.3781 (3)	0.1119 (5)	0.0402 (2)	0.0780 (16)	
H7CA	0.395409	0.135654	0.004936	0.094*	
H7CB	0.343097	0.031626	0.031706	0.094*	
C8C	0.4513 (3)	0.0795 (5)	0.0889 (2)	0.0778 (15)	
H8CA	0.433740	0.046830	0.122885	0.093*	
H8CB	0.482818	0.007113	0.076378	0.093*	
C9C	0.4560 (3)	0.3197 (6)	0.1201 (2)	0.0896 (18)	
H9CA	0.490449	0.400734	0.127955	0.107*	
H9CB	0.438091	0.298123	0.155397	0.107*	
C10C	0.3834 (3)	0.3483 (5)	0.0706 (3)	0.0967 (19)	
H10A	0.351600	0.422484	0.081479	0.116*	
H10B	0.401437	0.376935	0.036203	0.116*	
F1D	0.80958 (18)	0.2961 (4)	−0.15139 (13)	0.1174 (11)	
O3D	0.56389 (18)	0.2765 (4)	0.01163 (13)	0.0890 (12)	
O4D	0.68138 (17)	0.2812 (3)	0.07894 (14)	0.0732 (9)	
C11D	0.6858 (2)	0.2799 (4)	−0.02171 (19)	0.0491 (11)	
C12D	0.6447 (3)	0.2743 (5)	−0.0801 (2)	0.0695 (14)	
H12D	0.588034	0.266742	−0.090222	0.083*	
C13D	0.6866 (3)	0.2796 (5)	−0.1238 (2)	0.0842 (17)	
H13D	0.658864	0.276926	−0.163259	0.101*	
C14D	0.7692 (3)	0.2888 (5)	−0.1075 (2)	0.0737 (15)	
C15D	0.8119 (3)	0.2932 (5)	−0.0508 (2)	0.0707 (14)	
H15D	0.868565	0.299146	−0.041181	0.085*	
C16D	0.7698 (3)	0.2886 (5)	−0.0074 (2)	0.0629 (13)	
H16D	0.798341	0.291399	0.031866	0.075*	
C17D	0.6400 (3)	0.2786 (5)	0.0263 (2)	0.0595 (13)	
O2B	0.3231 (2)	0.2769 (5)	0.20682 (17)	0.1045 (14)	
O1B	0.3290 (2)	0.0563 (5)	0.20074 (18)	0.1105 (15)	
N1B	−0.0349 (2)	0.1235 (4)	0.21687 (19)	0.0790 (13)	
N2B	−0.2044 (2)	0.1456 (5)	0.16528 (17)	0.0588 (11)	
H2BA	−0.2443 (18)	0.172 (4)	0.1358 (13)	0.071*	
H2BB	−0.227 (2)	0.120 (4)	0.1942 (13)	0.071*	
N3B	0.2917 (3)	0.1638 (6)	0.20481 (18)	0.0761 (14)	
C1B	0.0441 (3)	0.1348 (5)	0.21270 (19)	0.0566 (13)	
C2B	0.0898 (3)	0.0181 (5)	0.2067 (2)	0.0733 (15)	
H2B	0.065666	−0.068307	0.204790	0.088*	
C3B	0.1698 (3)	0.0287 (6)	0.2037 (2)	0.0735 (15)	
H3B	0.199054	−0.050062	0.199530	0.088*	
C4B	0.2063 (3)	0.1544 (7)	0.20692 (19)	0.0605 (14)	
C5B	0.1634 (3)	0.2711 (5)	0.21249 (18)	0.0651 (14)	
H5B	0.188542	0.356672	0.214251	0.078*	
C6B	0.0836 (3)	0.2623 (5)	0.21548 (18)	0.0640 (14)	
H6B	0.055117	0.342280	0.219424	0.077*	
C7B	−0.0848 (3)	−0.0001 (5)	0.2001 (2)	0.0835 (16)	
H7BA	−0.104035	−0.032659	0.233468	0.100*	
H7BB	−0.051975	−0.072114	0.188784	0.100*	
C8B	−0.1567 (3)	0.0313 (5)	0.1496 (2)	0.0746 (15)	
H8BA	−0.137606	0.055819	0.115176	0.090*	
H8BB	−0.191039	−0.049624	0.140231	0.090*	
C9B	−0.1539 (3)	0.2701 (5)	0.1840 (2)	0.0664 (14)	
H9BA	−0.186574	0.340691	0.196546	0.080*	
H9BB	−0.134516	0.305835	0.151264	0.080*	
C10B	−0.0822 (3)	0.2335 (5)	0.2341 (2)	0.0730 (15)	
H10C	−0.047802	0.313746	0.245332	0.088*	
H10D	−0.101734	0.204666	0.267845	0.088*	
F1A	−0.49683 (18)	0.1699 (4)	0.43216 (14)	0.1397 (14)	
O4A	−0.38370 (18)	0.0984 (4)	0.19821 (14)	0.0888 (11)	
O3A	−0.26382 (17)	0.0937 (3)	0.26186 (12)	0.0729 (10)	
C11A	−0.3810 (2)	0.1255 (4)	0.29882 (19)	0.0477 (11)	
C16A	−0.4654 (3)	0.1210 (4)	0.2880 (2)	0.0600 (13)	
H16A	−0.496139	0.108480	0.249617	0.072*	
C15A	−0.5051 (3)	0.1346 (5)	0.3326 (2)	0.0745 (15)	
H15A	−0.561728	0.129803	0.325026	0.089*	
C14A	−0.4589 (3)	0.1549 (6)	0.3875 (2)	0.0849 (17)	
C13A	−0.3756 (3)	0.1629 (6)	0.4012 (2)	0.0885 (18)	
H13A	−0.345964	0.178267	0.439690	0.106*	
C12A	−0.3368 (3)	0.1473 (5)	0.3559 (2)	0.0666 (14)	
H12A	−0.280128	0.151696	0.364060	0.080*	
C17A	−0.3395 (3)	0.1061 (5)	0.2497 (2)	0.0585 (13)	
O5	0.6374 (2)	0.4752 (4)	0.15259 (17)	0.0930 (12)	
H5A	0.678 (2)	0.513 (5)	0.1756 (19)	0.112*	
H5D	0.653 (3)	0.419 (4)	0.131 (2)	0.112*	
O6	0.3427 (3)	0.0628 (6)	−0.1109 (2)	0.1407 (17)	
H6A	0.349 (4)	0.042 (7)	−0.1444 (15)	0.169*	
H6D	0.371 (4)	0.135 (4)	−0.106 (3)	0.169*	
O7	0.4318 (3)	0.3010 (5)	−0.0864 (3)	0.1502 (18)	
H7A	0.473 (3)	0.311 (7)	−0.059 (2)	0.180*	
H7B	0.414 (4)	0.373 (4)	−0.104 (3)	0.180*	

### Bis[4-(4-nitrophenyl)piperazin-1-ium] bis(4-fluorobenzoate) trihydrate (2) Atomic displacement parameters (Å^2^)

**Table:**

	*U*^11^	*U*^22^	*U*^33^	*U*^12^	*U*^13^	*U*^23^
O1C	0.077 (3)	0.203 (5)	0.119 (4)	−0.059 (3)	0.032 (2)	−0.048 (4)
O2C	0.064 (3)	0.187 (5)	0.111 (4)	0.008 (3)	0.025 (2)	−0.019 (4)
N1C	0.045 (3)	0.063 (3)	0.120 (4)	0.003 (2)	0.014 (2)	−0.015 (3)
N2C	0.043 (3)	0.123 (4)	0.060 (3)	0.001 (3)	0.012 (2)	0.013 (3)
N3C	0.062 (4)	0.165 (6)	0.060 (3)	−0.010 (4)	0.010 (3)	−0.029 (4)
C1C	0.041 (3)	0.065 (3)	0.052 (3)	−0.002 (3)	0.000 (2)	0.002 (3)
C2C	0.055 (3)	0.075 (4)	0.080 (4)	−0.008 (3)	0.012 (3)	−0.012 (3)
C3C	0.059 (4)	0.091 (5)	0.078 (4)	−0.026 (3)	0.011 (3)	−0.013 (3)
C4C	0.040 (3)	0.111 (5)	0.051 (3)	−0.003 (4)	0.007 (2)	−0.009 (4)
C5C	0.053 (3)	0.091 (5)	0.073 (4)	0.010 (3)	0.010 (3)	−0.003 (3)
C6C	0.049 (3)	0.065 (4)	0.088 (4)	0.003 (3)	0.013 (3)	0.000 (3)
C7C	0.052 (3)	0.088 (4)	0.090 (4)	0.003 (3)	0.011 (3)	−0.019 (3)
C8C	0.058 (3)	0.089 (4)	0.089 (4)	0.009 (3)	0.022 (3)	0.007 (3)
C9C	0.063 (4)	0.103 (5)	0.110 (5)	−0.023 (3)	0.035 (3)	−0.025 (4)
C10C	0.054 (3)	0.069 (4)	0.162 (6)	−0.001 (3)	0.017 (3)	0.004 (4)
F1D	0.107 (2)	0.179 (3)	0.084 (2)	−0.010 (2)	0.0579 (19)	−0.012 (2)
O3D	0.048 (2)	0.157 (3)	0.063 (2)	0.004 (2)	0.0152 (17)	0.019 (2)
O4D	0.065 (2)	0.100 (3)	0.054 (2)	0.0097 (18)	0.0136 (17)	0.009 (2)
C11D	0.045 (3)	0.053 (3)	0.050 (3)	0.000 (2)	0.014 (2)	0.005 (3)
C12D	0.056 (3)	0.090 (4)	0.059 (4)	−0.005 (3)	0.009 (3)	−0.008 (3)
C13D	0.084 (4)	0.124 (5)	0.048 (3)	−0.010 (4)	0.024 (3)	−0.017 (3)
C14D	0.073 (4)	0.090 (4)	0.074 (4)	−0.006 (3)	0.048 (3)	−0.008 (4)
C15D	0.058 (3)	0.085 (4)	0.069 (4)	0.003 (3)	0.016 (3)	0.002 (4)
C16D	0.058 (3)	0.080 (4)	0.054 (3)	0.004 (3)	0.019 (3)	0.011 (3)
C17D	0.056 (3)	0.072 (4)	0.050 (3)	0.004 (3)	0.012 (3)	0.008 (3)
O2B	0.073 (3)	0.147 (4)	0.101 (3)	−0.043 (3)	0.036 (2)	−0.035 (3)
O1B	0.055 (2)	0.145 (4)	0.134 (4)	0.022 (2)	0.028 (2)	0.016 (3)
N1B	0.041 (2)	0.065 (3)	0.131 (4)	−0.004 (2)	0.020 (2)	−0.014 (3)
N2B	0.047 (3)	0.074 (3)	0.056 (3)	0.008 (3)	0.0141 (19)	0.009 (3)
N3B	0.051 (3)	0.123 (5)	0.054 (3)	−0.006 (3)	0.012 (2)	0.001 (3)
C1B	0.039 (3)	0.065 (4)	0.059 (3)	0.007 (3)	−0.001 (2)	0.002 (3)
C2B	0.044 (3)	0.069 (4)	0.103 (4)	0.002 (3)	0.010 (3)	0.009 (3)
C3B	0.052 (3)	0.080 (4)	0.085 (4)	0.011 (3)	0.010 (3)	0.006 (3)
C4B	0.038 (3)	0.091 (4)	0.050 (3)	−0.008 (3)	0.005 (2)	0.002 (3)
C5B	0.060 (3)	0.078 (4)	0.054 (3)	−0.010 (3)	0.009 (2)	−0.005 (3)
C6B	0.053 (3)	0.069 (4)	0.068 (4)	−0.002 (3)	0.010 (2)	−0.010 (3)
C7B	0.049 (3)	0.066 (4)	0.137 (5)	0.002 (3)	0.025 (3)	0.003 (4)
C8B	0.057 (3)	0.074 (4)	0.100 (4)	−0.004 (3)	0.033 (3)	−0.017 (3)
C9B	0.060 (3)	0.060 (3)	0.081 (4)	0.004 (3)	0.021 (3)	0.001 (3)
C10B	0.052 (3)	0.082 (4)	0.086 (4)	0.004 (3)	0.018 (3)	−0.014 (3)
F1A	0.100 (2)	0.246 (4)	0.090 (2)	−0.018 (2)	0.055 (2)	−0.043 (3)
O4A	0.067 (2)	0.145 (3)	0.051 (2)	0.020 (2)	0.0076 (18)	−0.003 (2)
O3A	0.0438 (19)	0.116 (3)	0.061 (2)	0.0097 (18)	0.0175 (16)	0.012 (2)
C11A	0.042 (3)	0.052 (3)	0.048 (3)	0.001 (2)	0.010 (2)	0.000 (3)
C16A	0.055 (3)	0.070 (3)	0.054 (3)	−0.001 (3)	0.010 (2)	−0.005 (3)
C15A	0.055 (3)	0.102 (4)	0.070 (4)	−0.013 (3)	0.021 (3)	−0.018 (4)
C14A	0.068 (4)	0.131 (5)	0.069 (4)	−0.011 (4)	0.043 (3)	−0.023 (4)
C13A	0.075 (4)	0.141 (5)	0.050 (3)	−0.012 (4)	0.017 (3)	−0.024 (4)
C12A	0.055 (3)	0.090 (4)	0.053 (3)	−0.005 (3)	0.010 (3)	−0.009 (3)
C17A	0.055 (3)	0.072 (4)	0.050 (3)	0.007 (3)	0.016 (3)	0.007 (3)
O5	0.061 (2)	0.112 (3)	0.100 (3)	−0.002 (2)	0.008 (2)	−0.036 (3)
O6	0.115 (3)	0.170 (5)	0.149 (4)	−0.041 (3)	0.056 (3)	−0.068 (4)
O7	0.122 (4)	0.128 (4)	0.156 (5)	−0.024 (3)	−0.050 (3)	0.019 (4)

### Bis[4-(4-nitrophenyl)piperazin-1-ium] bis(4-fluorobenzoate) trihydrate (2) Geometric parameters (Å, º)

**Table:**

O1C—N3C	1.203 (6)	N1B—C10B	1.449 (5)
O2C—N3C	1.237 (6)	N1B—C7B	1.464 (5)
N1C—C1C	1.368 (5)	N2B—C8B	1.470 (5)
N1C—C7C	1.449 (5)	N2B—C9B	1.483 (5)
N1C—C10C	1.454 (5)	N2B—H2BA	0.875 (18)
N2C—C9C	1.474 (6)	N2B—H2BB	0.892 (18)
N2C—C8C	1.478 (6)	N3B—C4B	1.456 (6)
N2C—H2CA	0.897 (19)	C1B—C2B	1.398 (6)
N2C—H2CB	0.869 (19)	C1B—C6B	1.402 (5)
N3C—C4C	1.460 (6)	C2B—C3B	1.372 (5)
C1C—C2C	1.391 (5)	C2B—H2B	0.9300
C1C—C6C	1.391 (5)	C3B—C4B	1.363 (6)
C2C—C3C	1.382 (6)	C3B—H3B	0.9300
C2C—H2C	0.9300	C4B—C5B	1.369 (6)
C3C—C4C	1.368 (6)	C5B—C6B	1.369 (5)
C3C—H3C	0.9300	C5B—H5B	0.9300
C4C—C5C	1.354 (6)	C6B—H6B	0.9300
C5C—C6C	1.355 (5)	C7B—C8B	1.505 (5)
C5C—H5C	0.9300	C7B—H7BA	0.9700
C6C—H6C	0.9300	C7B—H7BB	0.9700
C7C—C8C	1.495 (5)	C8B—H8BA	0.9700
C7C—H7CA	0.9700	C8B—H8BB	0.9700
C7C—H7CB	0.9700	C9B—C10B	1.508 (5)
C8C—H8CA	0.9700	C9B—H9BA	0.9700
C8C—H8CB	0.9700	C9B—H9BB	0.9700
C9C—C10C	1.493 (6)	C10B—H10C	0.9700
C9C—H9CA	0.9700	C10B—H10D	0.9700
C9C—H9CB	0.9700	F1A—C14A	1.363 (5)
C10C—H10A	0.9700	O4A—C17A	1.257 (5)
C10C—H10B	0.9700	O3A—C17A	1.245 (4)
F1D—C14D	1.368 (5)	C11A—C12A	1.379 (5)
O3D—C17D	1.247 (4)	C11A—C16A	1.386 (5)
O4D—C17D	1.260 (5)	C11A—C17A	1.499 (6)
C11D—C12D	1.376 (5)	C16A—C15A	1.380 (6)
C11D—C16D	1.377 (5)	C16A—H16A	0.9300
C11D—C17D	1.514 (6)	C15A—C14A	1.346 (6)
C12D—C13D	1.381 (6)	C15A—H15A	0.9300
C12D—H12D	0.9300	C14A—C13A	1.365 (6)
C13D—C14D	1.356 (6)	C13A—C12A	1.387 (6)
C13D—H13D	0.9300	C13A—H13A	0.9300
C14D—C15D	1.350 (6)	C12A—H12A	0.9300
C15D—C16D	1.375 (6)	O5—H5A	0.843 (19)
C15D—H15D	0.9300	O5—H5D	0.833 (19)
C16D—H16D	0.9300	O6—H6A	0.84 (2)
O2B—N3B	1.216 (5)	O6—H6D	0.84 (2)
O1B—N3B	1.236 (5)	O7—H7A	0.83 (2)
N1B—C1B	1.365 (5)	O7—H7B	0.83 (2)
			
C1C—N1C—C7C	125.0 (4)	C10B—N1B—C7B	111.1 (4)
C1C—N1C—C10C	123.3 (4)	C8B—N2B—C9B	112.3 (3)
C7C—N1C—C10C	111.6 (4)	C8B—N2B—H2BA	113 (3)
C9C—N2C—C8C	111.6 (4)	C9B—N2B—H2BA	106 (3)
C9C—N2C—H2CA	109 (3)	C8B—N2B—H2BB	110 (3)
C8C—N2C—H2CA	111 (3)	C9B—N2B—H2BB	109 (3)
C9C—N2C—H2CB	113 (3)	H2BA—N2B—H2BB	107 (4)
C8C—N2C—H2CB	108 (3)	O2B—N3B—O1B	122.8 (5)
H2CA—N2C—H2CB	105 (4)	O2B—N3B—C4B	118.7 (5)
O1C—N3C—O2C	123.3 (6)	O1B—N3B—C4B	118.5 (5)
O1C—N3C—C4C	120.2 (6)	N1B—C1B—C2B	121.0 (5)
O2C—N3C—C4C	116.5 (6)	N1B—C1B—C6B	122.0 (5)
N1C—C1C—C2C	121.3 (5)	C2B—C1B—C6B	117.0 (4)
N1C—C1C—C6C	122.0 (5)	C3B—C2B—C1B	121.2 (5)
C2C—C1C—C6C	116.7 (4)	C3B—C2B—H2B	119.4
C3C—C2C—C1C	120.9 (5)	C1B—C2B—H2B	119.4
C3C—C2C—H2C	119.6	C4B—C3B—C2B	120.2 (5)
C1C—C2C—H2C	119.6	C4B—C3B—H3B	119.9
C4C—C3C—C2C	119.6 (5)	C2B—C3B—H3B	119.9
C4C—C3C—H3C	120.2	C3B—C4B—C5B	120.3 (4)
C2C—C3C—H3C	120.2	C3B—C4B—N3B	119.5 (6)
C5C—C4C—C3C	120.6 (5)	C5B—C4B—N3B	120.2 (5)
C5C—C4C—N3C	121.3 (6)	C6B—C5B—C4B	120.3 (5)
C3C—C4C—N3C	118.1 (6)	C6B—C5B—H5B	119.9
C4C—C5C—C6C	119.9 (5)	C4B—C5B—H5B	119.9
C4C—C5C—H5C	120.1	C5B—C6B—C1B	121.1 (5)
C6C—C5C—H5C	120.1	C5B—C6B—H6B	119.5
C5C—C6C—C1C	122.3 (5)	C1B—C6B—H6B	119.5
C5C—C6C—H6C	118.9	N1B—C7B—C8B	110.2 (4)
C1C—C6C—H6C	118.9	N1B—C7B—H7BA	109.6
N1C—C7C—C8C	110.3 (4)	C8B—C7B—H7BA	109.6
N1C—C7C—H7CA	109.6	N1B—C7B—H7BB	109.6
C8C—C7C—H7CA	109.6	C8B—C7B—H7BB	109.6
N1C—C7C—H7CB	109.6	H7BA—C7B—H7BB	108.1
C8C—C7C—H7CB	109.6	N2B—C8B—C7B	110.0 (4)
H7CA—C7C—H7CB	108.1	N2B—C8B—H8BA	109.7
N2C—C8C—C7C	110.4 (4)	C7B—C8B—H8BA	109.7
N2C—C8C—H8CA	109.6	N2B—C8B—H8BB	109.7
C7C—C8C—H8CA	109.6	C7B—C8B—H8BB	109.7
N2C—C8C—H8CB	109.6	H8BA—C8B—H8BB	108.2
C7C—C8C—H8CB	109.6	N2B—C9B—C10B	109.4 (4)
H8CA—C8C—H8CB	108.1	N2B—C9B—H9BA	109.8
N2C—C9C—C10C	110.1 (4)	C10B—C9B—H9BA	109.8
N2C—C9C—H9CA	109.6	N2B—C9B—H9BB	109.8
C10C—C9C—H9CA	109.6	C10B—C9B—H9BB	109.8
N2C—C9C—H9CB	109.6	H9BA—C9B—H9BB	108.2
C10C—C9C—H9CB	109.6	N1B—C10B—C9B	110.4 (4)
H9CA—C9C—H9CB	108.2	N1B—C10B—H10C	109.6
N1C—C10C—C9C	110.2 (4)	C9B—C10B—H10C	109.6
N1C—C10C—H10A	109.6	N1B—C10B—H10D	109.6
C9C—C10C—H10A	109.6	C9B—C10B—H10D	109.6
N1C—C10C—H10B	109.6	H10C—C10B—H10D	108.1
C9C—C10C—H10B	109.6	C12A—C11A—C16A	118.0 (4)
H10A—C10C—H10B	108.1	C12A—C11A—C17A	121.4 (4)
C12D—C11D—C16D	118.9 (4)	C16A—C11A—C17A	120.5 (4)
C12D—C11D—C17D	120.9 (4)	C15A—C16A—C11A	121.8 (4)
C16D—C11D—C17D	120.2 (4)	C15A—C16A—H16A	119.1
C11D—C12D—C13D	120.7 (4)	C11A—C16A—H16A	119.1
C11D—C12D—H12D	119.7	C14A—C15A—C16A	117.6 (5)
C13D—C12D—H12D	119.7	C14A—C15A—H15A	121.2
C14D—C13D—C12D	118.2 (5)	C16A—C15A—H15A	121.2
C14D—C13D—H13D	120.9	C15A—C14A—F1A	118.7 (5)
C12D—C13D—H13D	120.9	C15A—C14A—C13A	123.8 (5)
C15D—C14D—C13D	122.9 (5)	F1A—C14A—C13A	117.6 (5)
C15D—C14D—F1D	119.7 (5)	C14A—C13A—C12A	117.7 (5)
C13D—C14D—F1D	117.4 (5)	C14A—C13A—H13A	121.1
C14D—C15D—C16D	118.7 (5)	C12A—C13A—H13A	121.1
C14D—C15D—H15D	120.7	C11A—C12A—C13A	121.1 (4)
C16D—C15D—H15D	120.7	C11A—C12A—H12A	119.5
C15D—C16D—C11D	120.6 (4)	C13A—C12A—H12A	119.5
C15D—C16D—H16D	119.7	O3A—C17A—O4A	123.3 (4)
C11D—C16D—H16D	119.7	O3A—C17A—C11A	118.9 (4)
O3D—C17D—O4D	123.9 (4)	O4A—C17A—C11A	117.8 (4)
O3D—C17D—C11D	118.3 (4)	H5A—O5—H5D	110 (5)
O4D—C17D—C11D	117.8 (4)	H6A—O6—H6D	98 (7)
C1B—N1B—C10B	125.1 (4)	H7A—O7—H7B	115 (7)
C1B—N1B—C7B	123.7 (4)		
			
C7C—N1C—C1C—C2C	8.1 (7)	C10B—N1B—C1B—C2B	167.7 (4)
C10C—N1C—C1C—C2C	−175.0 (5)	C7B—N1B—C1B—C2B	−17.0 (7)
C7C—N1C—C1C—C6C	−172.0 (4)	C10B—N1B—C1B—C6B	−10.8 (7)
C10C—N1C—C1C—C6C	4.9 (7)	C7B—N1B—C1B—C6B	164.5 (5)
N1C—C1C—C2C—C3C	178.4 (4)	N1B—C1B—C2B—C3B	−178.7 (4)
C6C—C1C—C2C—C3C	−1.6 (7)	C6B—C1B—C2B—C3B	−0.1 (7)
C1C—C2C—C3C—C4C	0.3 (7)	C1B—C2B—C3B—C4B	0.4 (7)
C2C—C3C—C4C—C5C	1.3 (8)	C2B—C3B—C4B—C5B	−0.7 (7)
C2C—C3C—C4C—N3C	−178.2 (4)	C2B—C3B—C4B—N3B	178.8 (4)
O1C—N3C—C4C—C5C	−179.5 (5)	O2B—N3B—C4B—C3B	178.9 (5)
O2C—N3C—C4C—C5C	−0.4 (7)	O1B—N3B—C4B—C3B	−0.7 (7)
O1C—N3C—C4C—C3C	−0.1 (7)	O2B—N3B—C4B—C5B	−1.6 (7)
O2C—N3C—C4C—C3C	179.0 (5)	O1B—N3B—C4B—C5B	178.8 (5)
C3C—C4C—C5C—C6C	−1.4 (7)	C3B—C4B—C5B—C6B	0.6 (7)
N3C—C4C—C5C—C6C	178.0 (4)	N3B—C4B—C5B—C6B	−178.9 (4)
C4C—C5C—C6C—C1C	0.0 (7)	C4B—C5B—C6B—C1B	−0.3 (7)
N1C—C1C—C6C—C5C	−178.5 (4)	N1B—C1B—C6B—C5B	178.6 (4)
C2C—C1C—C6C—C5C	1.4 (7)	C2B—C1B—C6B—C5B	0.0 (6)
C1C—N1C—C7C—C8C	−124.1 (5)	C1B—N1B—C7B—C8B	−116.6 (5)
C10C—N1C—C7C—C8C	58.7 (5)	C10B—N1B—C7B—C8B	59.3 (5)
C9C—N2C—C8C—C7C	55.0 (5)	C9B—N2B—C8B—C7B	55.5 (5)
N1C—C7C—C8C—N2C	−55.7 (5)	N1B—C7B—C8B—N2B	−56.2 (5)
C8C—N2C—C9C—C10C	−55.5 (5)	C8B—N2B—C9B—C10B	−55.5 (5)
C1C—N1C—C10C—C9C	123.4 (5)	C1B—N1B—C10B—C9B	116.0 (5)
C7C—N1C—C10C—C9C	−59.4 (6)	C7B—N1B—C10B—C9B	−59.8 (5)
N2C—C9C—C10C—N1C	56.9 (6)	N2B—C9B—C10B—N1B	56.8 (5)
C16D—C11D—C12D—C13D	−1.1 (8)	C12A—C11A—C16A—C15A	−1.5 (7)
C17D—C11D—C12D—C13D	178.1 (5)	C17A—C11A—C16A—C15A	178.1 (5)
C11D—C12D—C13D—C14D	0.8 (8)	C11A—C16A—C15A—C14A	1.1 (8)
C12D—C13D—C14D—C15D	−0.1 (9)	C16A—C15A—C14A—F1A	179.4 (5)
C12D—C13D—C14D—F1D	−179.0 (5)	C16A—C15A—C14A—C13A	0.1 (9)
C13D—C14D—C15D—C16D	−0.3 (9)	C15A—C14A—C13A—C12A	−0.8 (9)
F1D—C14D—C15D—C16D	178.6 (4)	F1A—C14A—C13A—C12A	179.8 (5)
C14D—C15D—C16D—C11D	−0.1 (8)	C16A—C11A—C12A—C13A	0.6 (7)
C12D—C11D—C16D—C15D	0.8 (7)	C17A—C11A—C12A—C13A	−178.9 (5)
C17D—C11D—C16D—C15D	−178.5 (5)	C14A—C13A—C12A—C11A	0.4 (8)
C12D—C11D—C17D—O3D	−2.2 (7)	C12A—C11A—C17A—O3A	8.3 (7)
C16D—C11D—C17D—O3D	177.0 (4)	C16A—C11A—C17A—O3A	−171.2 (4)
C12D—C11D—C17D—O4D	178.6 (4)	C12A—C11A—C17A—O4A	−174.1 (4)
C16D—C11D—C17D—O4D	−2.2 (7)	C16A—C11A—C17A—O4A	6.4 (7)

### Bis[4-(4-nitrophenyl)piperazin-1-ium] bis(4-fluorobenzoate) trihydrate (2) Hydrogen-bond geometry (Å, º)

**Table:**

*D*—H···*A*	*D*—H	H···*A*	*D*···*A*	*D*—H···*A*
N2*C*—H2*CA*···O3*D*	0.90 (2)	1.84 (2)	2.733 (5)	176 (4)
N2*C*—H2*CB*···O4*A*^i^	0.87 (2)	1.87 (2)	2.726 (5)	166 (4)
C3*C*—H3*C*···O1*C*^ii^	0.93	2.62	3.261 (7)	126
C9*C*—H9*CA*···O5	0.97	2.51	3.332 (6)	142
C9*C*—H9*CB*···O2*B*	0.97	2.53	3.401 (6)	149
C15*D*—H15*D*···F1*A*^iii^	0.93	2.52	3.369 (5)	152
N2*B*—H2*BA*···O4*D*^iv^	0.88 (2)	1.92 (2)	2.764 (5)	163 (4)
N2*B*—H2*BB*···O3*A*	0.89 (2)	1.86 (2)	2.739 (5)	169 (4)
C3*B*—H3*B*···F1*D*^v^	0.93	2.63	3.436 (6)	145
C8*B*—H8*BA*···O1*C*	0.97	2.53	3.437 (6)	155
C8*B*—H8*BB*···O6^ii^	0.97	2.49	3.182 (6)	129
C15*A*—H15*A*···F1*D*^vi^	0.93	2.48	3.310 (5)	149
O5—H5*A*···O3*A*^vii^	0.84 (2)	1.96 (2)	2.794 (4)	172 (5)
O5—H5*D*···O4*D*	0.83 (2)	1.95 (2)	2.778 (5)	175 (5)
O6—H6*A*···O4*A*^ii^	0.84 (2)	2.04 (4)	2.795 (6)	149 (7)
O6—H6*D*···O7	0.84 (2)	1.91 (2)	2.744 (7)	174 (8)
O7—H7*A*···O3*D*	0.83 (2)	1.99 (3)	2.794 (5)	163 (7)
O7—H7*B*···O5^viii^	0.83 (2)	1.93 (2)	2.759 (6)	172 (7)

Symmetry codes: (i) *x*+1, *y*, *z*; (ii) −*x*, −*y*, −*z*; (iii) *x*+3/2, −*y*+1/2, *z*−1/2; (iv) *x*−1, *y*, *z*; (v) −*x*+1, −*y*, −*z*; (vi) *x*−3/2, −*y*+1/2, *z*+1/2; (vii) −*x*+1/2, *y*+1/2, −*z*+1/2; (viii) −*x*+1, −*y*+1, −*z*.

### 4-(4-Nitrophenyl)piperazin-1-ium 3,5-dinitrobenzoate (3) Crystal data

**Table:**

C_10_H_14_N_3_O_2_^+^·C_7_H_3_N_2_O_6_^−^	*F*(000) = 1744
*M**_r_* = 419.35	*D*_x_ = 1.443 Mg m^−^^3^
Monoclinic, *C*2/*c*	Mo *K*α radiation, λ = 0.71073 Å
*a* = 27.953 (6) Å	Cell parameters from 4154 reflections
*b* = 8.1422 (6) Å	θ = 2.6–27.7°
*c* = 24.657 (5) Å	µ = 0.12 mm^−^^1^
β = 136.55 (4)°	*T* = 293 K
*V* = 3859 (2) Å^3^	Prism, orange
*Z* = 8	0.48 × 0.48 × 0.40 mm

### 4-(4-Nitrophenyl)piperazin-1-ium 3,5-dinitrobenzoate (3) Data collection

**Table:**

Oxford Diffraction Xcalibur with Sapphire CCD diffractometer	2838 reflections with *I* > 2σ(*I*)
Radiation source: Enhance (Mo) X-ray Source	*R*_int_ = 0.015
Rotation method data acquisition using ω scans.	θ_max_ = 27.7°, θ_min_ = 2.6°
Absorption correction: multi-scan (CrysAlis RED; Oxford Diffraction, 2009)	*h* = −35→23
*T*_min_ = 0.621, *T*_max_ = 1.000	*k* = −10→10
8107 measured reflections	*l* = −31→30
4119 independent reflections	

### 4-(4-Nitrophenyl)piperazin-1-ium 3,5-dinitrobenzoate (3) Refinement

**Table:**

Refinement on *F*^2^	Primary atom site location: dual
Least-squares matrix: full	Secondary atom site location: difference Fourier map
*R*[*F*^2^ > 2σ(*F*^2^)] = 0.044	Hydrogen site location: mixed
*wR*(*F*^2^) = 0.119	H atoms treated by a mixture of independent and constrained refinement
*S* = 1.03	*w* = 1/[σ^2^(*F*_o_^2^) + (0.0543*P*)^2^ + 1.7099*P*] where *P* = (*F*_o_^2^ + 2*F*_c_^2^)/3
4119 reflections	(Δ/σ)_max_ < 0.001
337 parameters	Δρ_max_ = 0.24 e Å^−^^3^
262 restraints	Δρ_min_ = −0.24 e Å^−^^3^

### 4-(4-Nitrophenyl)piperazin-1-ium 3,5-dinitrobenzoate (3) Special details

**Table:**

Geometry. All esds (except the esd in the dihedral angle between two l.s. planes) are estimated using the full covariance matrix. The cell esds are taken into account individually in the estimation of esds in distances, angles and torsion angles; correlations between esds in cell parameters are only used when they are defined by crystal symmetry. An approximate (isotropic) treatment of cell esds is used for estimating esds involving l.s. planes.

### 4-(4-Nitrophenyl)piperazin-1-ium 3,5-dinitrobenzoate (3) Fractional atomic coordinates and isotropic or equivalent isotropic displacement parameters (Å^2^)

**Table:**

	*x*	*y*	*z*	*U*_iso_*/*U*_eq_	Occ. (<1)
N1	0.02612 (8)	0.1284 (2)	0.33993 (9)	0.0580 (4)	
N2	0.02315 (7)	−0.0150 (2)	0.44281 (9)	0.0452 (4)	
H21	−0.0246 (11)	−0.041 (2)	0.4078 (11)	0.054 (5)*	
H22	0.0488 (10)	−0.044 (2)	0.4941 (13)	0.060 (6)*	
O1	0.29131 (19)	0.2691 (7)	0.4152 (3)	0.0936 (12)	0.806 (10)
O2	0.28193 (17)	0.5004 (6)	0.4502 (2)	0.0809 (12)	0.806 (10)
N3	0.26170 (14)	0.3612 (7)	0.42375 (18)	0.0631 (11)	0.806 (10)
C1	0.08449 (14)	0.1834 (4)	0.3603 (2)	0.0484 (8)	0.806 (10)
C2	0.12478 (14)	0.0767 (5)	0.36260 (19)	0.0547 (7)	0.806 (10)
H2A	0.112973	−0.033824	0.350722	0.066*	0.806 (10)
C3	0.18272 (12)	0.1354 (5)	0.38267 (16)	0.0554 (8)	0.806 (10)
H3A	0.209682	0.064018	0.384227	0.067*	0.806 (10)
C4	0.20037 (11)	0.3006 (5)	0.40042 (16)	0.0508 (8)	0.806 (10)
C5	0.16008 (13)	0.4073 (4)	0.3981 (2)	0.0621 (8)	0.806 (10)
H5A	0.171890	0.517846	0.409976	0.075*	0.806 (10)
C6	0.10214 (14)	0.3487 (4)	0.3780 (2)	0.0613 (8)	0.806 (10)
H6A	0.075181	0.420007	0.376471	0.074*	0.806 (10)
O1A	0.2969 (7)	0.342 (2)	0.4346 (10)	0.082 (3)	0.194 (10)
O2A	0.2788 (8)	0.575 (2)	0.4647 (10)	0.092 (4)	0.194 (10)
N3A	0.2640 (8)	0.436 (2)	0.4386 (12)	0.067 (2)	0.194 (10)
C1A	0.0900 (6)	0.2138 (18)	0.3708 (9)	0.0517 (19)	0.194 (10)
C2A	0.1283 (6)	0.1262 (16)	0.3643 (9)	0.0519 (18)	0.194 (10)
H2AA	0.116265	0.018417	0.346160	0.062*	0.194 (10)
C3A	0.1845 (6)	0.1997 (19)	0.3848 (7)	0.0519 (17)	0.194 (10)
H3AA	0.210172	0.141125	0.380427	0.062*	0.194 (10)
C4A	0.2025 (5)	0.3609 (18)	0.4119 (7)	0.0608 (19)	0.194 (10)
C5A	0.1642 (6)	0.4485 (14)	0.4184 (8)	0.064 (2)	0.194 (10)
H5AA	0.176195	0.556282	0.436505	0.077*	0.194 (10)
C6A	0.1079 (6)	0.3750 (17)	0.3979 (9)	0.062 (2)	0.194 (10)
H6AA	0.082288	0.433576	0.402238	0.075*	0.194 (10)
C7	0.01491 (10)	−0.0473 (3)	0.33759 (11)	0.0549 (5)	
H7A	0.033495	−0.105603	0.321795	0.066*	
H7B	−0.034605	−0.069484	0.298825	0.066*	
C8	0.04986 (8)	−0.1082 (2)	0.41735 (9)	0.0453 (4)	
H8A	0.040452	−0.224338	0.414378	0.054*	
H8B	0.099796	−0.093615	0.455510	0.054*	
C9	0.02899 (9)	0.1652 (3)	0.44076 (12)	0.0566 (5)	
H9A	0.077636	0.196839	0.480671	0.068*	
H9B	0.006349	0.220270	0.452198	0.068*	
C10	−0.00458 (10)	0.2165 (3)	0.36060 (13)	0.0665 (6)	
H10A	−0.054078	0.193843	0.321442	0.080*	
H10B	0.001467	0.333748	0.360480	0.080*	
O3	−0.34048 (7)	0.22513 (19)	0.30638 (8)	0.0637 (4)	
O4	−0.43333 (6)	0.11002 (19)	0.19876 (9)	0.0728 (4)	
O5	−0.43221 (7)	−0.0409 (2)	0.00913 (7)	0.0831 (5)	
O6	−0.33493 (8)	−0.1049 (2)	0.05113 (8)	0.0724 (4)	
O7	−0.11517 (5)	0.10834 (17)	0.40386 (6)	0.0513 (3)	
O8	−0.11592 (5)	−0.06375 (17)	0.33293 (7)	0.0543 (3)	
N4	−0.37128 (7)	0.14524 (19)	0.24700 (10)	0.0500 (4)	
N5	−0.36882 (8)	−0.05526 (19)	0.06164 (8)	0.0563 (4)	
C11	−0.22389 (7)	0.03229 (19)	0.27718 (9)	0.0358 (4)	
C12	−0.26050 (8)	0.0878 (2)	0.29183 (9)	0.0379 (4)	
H12	−0.237068	0.124431	0.341817	0.046*	
C13	−0.33255 (8)	0.0880 (2)	0.23089 (10)	0.0411 (4)	
C14	−0.36957 (8)	0.0396 (2)	0.15541 (10)	0.0457 (4)	
H14	−0.417939	0.040358	0.115159	0.055*	
C15	−0.33151 (8)	−0.0098 (2)	0.14243 (9)	0.0432 (4)	
C16	−0.25940 (8)	−0.0165 (2)	0.20176 (9)	0.0398 (4)	
H16	−0.235352	−0.053046	0.191140	0.048*	
C17	−0.14489 (7)	0.0247 (2)	0.34377 (9)	0.0376 (4)	

### 4-(4-Nitrophenyl)piperazin-1-ium 3,5-dinitrobenzoate (3) Atomic displacement parameters (Å^2^)

**Table:**

	*U*^11^	*U*^22^	*U*^33^	*U*^12^	*U*^13^	*U*^23^
N1	0.0508 (8)	0.0655 (11)	0.0574 (9)	−0.0030 (8)	0.0391 (8)	0.0125 (8)
N2	0.0288 (7)	0.0606 (10)	0.0318 (7)	−0.0051 (7)	0.0173 (6)	0.0001 (7)
O1	0.0676 (16)	0.116 (3)	0.112 (2)	−0.0083 (18)	0.0703 (18)	−0.002 (2)
O2	0.0652 (15)	0.090 (3)	0.0628 (17)	−0.0371 (19)	0.0384 (14)	−0.006 (2)
N3	0.0402 (13)	0.088 (3)	0.0429 (14)	−0.0095 (16)	0.0242 (12)	0.0118 (16)
C1	0.0460 (11)	0.0555 (16)	0.0392 (12)	−0.0086 (10)	0.0294 (10)	0.0044 (11)
C2	0.0532 (11)	0.0565 (18)	0.0525 (11)	−0.0103 (12)	0.0378 (10)	−0.0025 (13)
C3	0.0523 (11)	0.0624 (19)	0.0520 (11)	−0.0039 (13)	0.0380 (10)	0.0016 (13)
C4	0.0438 (10)	0.061 (2)	0.0415 (11)	−0.0109 (11)	0.0291 (9)	0.0037 (12)
C5	0.0620 (12)	0.0578 (17)	0.0624 (15)	−0.0131 (12)	0.0438 (12)	−0.0005 (13)
C6	0.0606 (12)	0.0606 (17)	0.0696 (18)	−0.0051 (12)	0.0495 (13)	0.0016 (14)
O1A	0.045 (4)	0.091 (8)	0.075 (6)	−0.005 (5)	0.032 (4)	0.013 (6)
O2A	0.061 (5)	0.068 (6)	0.074 (7)	−0.026 (5)	0.025 (4)	0.004 (5)
N3A	0.049 (3)	0.065 (4)	0.053 (3)	−0.013 (3)	0.026 (3)	0.008 (3)
C1A	0.054 (2)	0.060 (3)	0.050 (2)	−0.007 (2)	0.0401 (18)	0.000 (2)
C2A	0.049 (2)	0.058 (3)	0.051 (2)	−0.013 (2)	0.0370 (17)	0.001 (2)
C3A	0.0483 (18)	0.060 (3)	0.053 (2)	−0.010 (2)	0.0386 (16)	0.006 (2)
C4A	0.0518 (19)	0.060 (3)	0.052 (2)	−0.012 (2)	0.0315 (17)	0.006 (2)
C5A	0.060 (2)	0.062 (3)	0.054 (2)	−0.014 (2)	0.0357 (19)	0.000 (2)
C6A	0.060 (2)	0.058 (3)	0.056 (3)	−0.014 (2)	0.0379 (19)	0.000 (2)
C7	0.0513 (10)	0.0639 (13)	0.0459 (10)	−0.0144 (9)	0.0341 (9)	−0.0059 (9)
C8	0.0346 (8)	0.0487 (10)	0.0395 (9)	−0.0048 (7)	0.0227 (7)	−0.0014 (8)
C9	0.0436 (9)	0.0572 (12)	0.0699 (13)	−0.0088 (9)	0.0415 (10)	−0.0121 (10)
C10	0.0492 (10)	0.0591 (13)	0.0887 (15)	0.0088 (10)	0.0493 (11)	0.0230 (11)
O3	0.0575 (8)	0.0796 (10)	0.0590 (8)	0.0076 (7)	0.0439 (7)	−0.0009 (8)
O4	0.0396 (7)	0.0735 (10)	0.0930 (11)	0.0004 (7)	0.0441 (8)	−0.0027 (8)
O5	0.0490 (8)	0.0826 (11)	0.0350 (7)	0.0164 (7)	0.0035 (6)	−0.0085 (7)
O6	0.0707 (9)	0.0865 (11)	0.0444 (8)	−0.0093 (8)	0.0367 (7)	−0.0170 (7)
O7	0.0331 (6)	0.0708 (9)	0.0299 (6)	−0.0012 (6)	0.0163 (5)	−0.0073 (6)
O8	0.0301 (6)	0.0648 (8)	0.0530 (7)	−0.0033 (6)	0.0253 (6)	−0.0166 (6)
N4	0.0391 (8)	0.0462 (9)	0.0597 (10)	0.0086 (7)	0.0341 (8)	0.0102 (8)
N5	0.0507 (9)	0.0451 (9)	0.0328 (8)	0.0016 (7)	0.0172 (7)	−0.0044 (7)
C11	0.0291 (7)	0.0349 (9)	0.0316 (8)	0.0000 (6)	0.0182 (6)	0.0014 (7)
C12	0.0327 (7)	0.0384 (9)	0.0313 (8)	0.0006 (7)	0.0195 (7)	0.0015 (7)
C13	0.0327 (8)	0.0366 (9)	0.0433 (9)	0.0042 (7)	0.0241 (7)	0.0044 (7)
C14	0.0273 (7)	0.0395 (9)	0.0396 (9)	0.0024 (7)	0.0143 (7)	0.0008 (7)
C15	0.0360 (8)	0.0366 (9)	0.0284 (8)	0.0008 (7)	0.0140 (7)	−0.0017 (7)
C16	0.0351 (8)	0.0393 (9)	0.0332 (8)	0.0012 (7)	0.0209 (7)	−0.0004 (7)
C17	0.0291 (7)	0.0422 (9)	0.0319 (8)	−0.0022 (7)	0.0190 (7)	0.0014 (7)

### 4-(4-Nitrophenyl)piperazin-1-ium 3,5-dinitrobenzoate (3) Geometric parameters (Å, º)

**Table:**

N1—C1	1.388 (3)	C5A—C6A	1.3900
N1—C7	1.457 (3)	C5A—H5AA	0.9300
N1—C10	1.465 (3)	C6A—H6AA	0.9300
N1—C1A	1.508 (9)	C7—C8	1.509 (3)
N2—C9	1.481 (3)	C7—H7A	0.9700
N2—C8	1.486 (2)	C7—H7B	0.9700
N2—H21	0.95 (2)	C8—H8A	0.9700
N2—H22	0.92 (2)	C8—H8B	0.9700
O1—N3	1.243 (4)	C9—C10	1.506 (3)
O2—N3	1.222 (4)	C9—H9A	0.9700
N3—C4	1.443 (3)	C9—H9B	0.9700
C1—C2	1.3900	C10—H10A	0.9700
C1—C6	1.3900	C10—H10B	0.9700
C2—C3	1.3900	O3—N4	1.216 (2)
C2—H2A	0.9300	O4—N4	1.229 (2)
C3—C4	1.3900	O5—N5	1.224 (2)
C3—H3A	0.9300	O6—N5	1.219 (2)
C4—C5	1.3900	O7—C17	1.250 (2)
C5—C6	1.3900	O8—C17	1.2491 (19)
C5—H5A	0.9300	N4—C13	1.473 (2)
C6—H6A	0.9300	N5—C15	1.475 (2)
O1A—N3A	1.251 (16)	C11—C12	1.385 (2)
O2A—N3A	1.217 (15)	C11—C16	1.386 (2)
N3A—C4A	1.455 (12)	C11—C17	1.520 (2)
C1A—C2A	1.3900	C12—C13	1.386 (2)
C1A—C6A	1.3900	C12—H12	0.9300
C2A—C3A	1.3900	C13—C14	1.376 (2)
C2A—H2AA	0.9300	C14—C15	1.375 (3)
C3A—C4A	1.3900	C14—H14	0.9300
C3A—H3AA	0.9300	C15—C16	1.387 (2)
C4A—C5A	1.3900	C16—H16	0.9300
			
C1—N1—C7	119.6 (2)	C1A—C6A—H6AA	120.0
C1—N1—C10	123.3 (2)	N1—C7—C8	110.72 (15)
C7—N1—C10	109.32 (15)	N1—C7—H7A	109.5
C7—N1—C1A	127.9 (6)	C8—C7—H7A	109.5
C10—N1—C1A	112.1 (6)	N1—C7—H7B	109.5
C9—N2—C8	112.95 (14)	C8—C7—H7B	109.5
C9—N2—H21	108.0 (12)	H7A—C7—H7B	108.1
C8—N2—H21	107.8 (11)	N2—C8—C7	109.52 (15)
C9—N2—H22	108.0 (12)	N2—C8—H8A	109.8
C8—N2—H22	108.5 (12)	C7—C8—H8A	109.8
H21—N2—H22	111.6 (17)	N2—C8—H8B	109.8
O2—N3—O1	122.9 (3)	C7—C8—H8B	109.8
O2—N3—C4	119.0 (3)	H8A—C8—H8B	108.2
O1—N3—C4	118.1 (3)	N2—C9—C10	109.91 (17)
N1—C1—C2	121.22 (19)	N2—C9—H9A	109.7
N1—C1—C6	118.78 (19)	C10—C9—H9A	109.7
C2—C1—C6	120.0	N2—C9—H9B	109.7
C3—C2—C1	120.0	C10—C9—H9B	109.7
C3—C2—H2A	120.0	H9A—C9—H9B	108.2
C1—C2—H2A	120.0	N1—C10—C9	110.88 (16)
C2—C3—C4	120.0	N1—C10—H10A	109.5
C2—C3—H3A	120.0	C9—C10—H10A	109.5
C4—C3—H3A	120.0	N1—C10—H10B	109.5
C5—C4—C3	120.0	C9—C10—H10B	109.5
C5—C4—N3	119.7 (2)	H10A—C10—H10B	108.1
C3—C4—N3	120.3 (2)	O3—N4—O4	123.95 (17)
C4—C5—C6	120.0	O3—N4—C13	118.51 (14)
C4—C5—H5A	120.0	O4—N4—C13	117.53 (17)
C6—C5—H5A	120.0	O6—N5—O5	123.96 (17)
C5—C6—C1	120.0	O6—N5—C15	118.28 (15)
C5—C6—H6A	120.0	O5—N5—C15	117.76 (19)
C1—C6—H6A	120.0	C12—C11—C16	119.88 (14)
O2A—N3A—O1A	128.2 (14)	C12—C11—C17	120.03 (14)
O2A—N3A—C4A	117.8 (13)	C16—C11—C17	120.08 (15)
O1A—N3A—C4A	114.0 (13)	C11—C12—C13	119.02 (15)
C2A—C1A—C6A	120.0	C11—C12—H12	120.5
C2A—C1A—N1	115.7 (8)	C13—C12—H12	120.5
C6A—C1A—N1	124.0 (8)	C14—C13—C12	122.68 (17)
C1A—C2A—C3A	120.0	C14—C13—N4	118.49 (15)
C1A—C2A—H2AA	120.0	C12—C13—N4	118.82 (16)
C3A—C2A—H2AA	120.0	C15—C14—C13	116.72 (15)
C4A—C3A—C2A	120.0	C15—C14—H14	121.6
C4A—C3A—H3AA	120.0	C13—C14—H14	121.6
C2A—C3A—H3AA	120.0	C14—C15—C16	122.91 (16)
C3A—C4A—C5A	120.0	C14—C15—N5	118.67 (15)
C3A—C4A—N3A	120.6 (8)	C16—C15—N5	118.41 (17)
C5A—C4A—N3A	119.3 (8)	C11—C16—C15	118.74 (16)
C6A—C5A—C4A	120.0	C11—C16—H16	120.6
C6A—C5A—H5AA	120.0	C15—C16—H16	120.6
C4A—C5A—H5AA	120.0	O8—C17—O7	126.27 (14)
C5A—C6A—C1A	120.0	O8—C17—C11	116.84 (14)
C5A—C6A—H6AA	120.0	O7—C17—C11	116.90 (15)
			
C7—N1—C1—C2	13.5 (3)	N1—C1A—C6A—C5A	−174.0 (12)
C10—N1—C1—C2	159.33 (19)	C1—N1—C7—C8	88.7 (2)
C7—N1—C1—C6	−166.94 (18)	C10—N1—C7—C8	−61.5 (2)
C10—N1—C1—C6	−21.1 (3)	C1A—N1—C7—C8	79.6 (8)
N1—C1—C2—C3	179.6 (3)	C9—N2—C8—C7	−53.44 (19)
C6—C1—C2—C3	0.0	N1—C7—C8—N2	57.24 (19)
C1—C2—C3—C4	0.0	C8—N2—C9—C10	53.10 (19)
C2—C3—C4—C5	0.0	C1—N1—C10—C9	−87.8 (3)
C2—C3—C4—N3	178.0 (2)	C7—N1—C10—C9	61.0 (2)
O2—N3—C4—C5	8.8 (4)	C1A—N1—C10—C9	−86.7 (7)
O1—N3—C4—C5	−170.6 (3)	N2—C9—C10—N1	−56.3 (2)
O2—N3—C4—C3	−169.2 (3)	C16—C11—C12—C13	2.3 (2)
O1—N3—C4—C3	11.4 (4)	C17—C11—C12—C13	−177.66 (15)
C3—C4—C5—C6	0.0	C11—C12—C13—C14	−2.0 (3)
N3—C4—C5—C6	−178.0 (2)	C11—C12—C13—N4	179.25 (15)
C4—C5—C6—C1	0.0	O3—N4—C13—C14	−161.90 (16)
N1—C1—C6—C5	−179.6 (3)	O4—N4—C13—C14	17.5 (2)
C2—C1—C6—C5	0.0	O3—N4—C13—C12	16.9 (2)
C7—N1—C1A—C2A	30.4 (11)	O4—N4—C13—C12	−163.67 (16)
C10—N1—C1A—C2A	170.6 (5)	C12—C13—C14—C15	−0.1 (3)
C7—N1—C1A—C6A	−155.4 (6)	N4—C13—C14—C15	178.72 (15)
C10—N1—C1A—C6A	−15.1 (10)	C13—C14—C15—C16	1.9 (3)
C6A—C1A—C2A—C3A	0.0	C13—C14—C15—N5	−177.15 (15)
N1—C1A—C2A—C3A	174.5 (11)	O6—N5—C15—C14	−177.23 (17)
C1A—C2A—C3A—C4A	0.0	O5—N5—C15—C14	2.7 (3)
C2A—C3A—C4A—C5A	0.0	O6—N5—C15—C16	3.7 (3)
C2A—C3A—C4A—N3A	175.8 (13)	O5—N5—C15—C16	−176.40 (17)
O2A—N3A—C4A—C3A	−177.1 (15)	C12—C11—C16—C15	−0.6 (2)
O1A—N3A—C4A—C3A	0 (2)	C17—C11—C16—C15	179.33 (15)
O2A—N3A—C4A—C5A	−1 (2)	C14—C15—C16—C11	−1.5 (3)
O1A—N3A—C4A—C5A	175.8 (13)	N5—C15—C16—C11	177.46 (15)
C3A—C4A—C5A—C6A	0.0	C12—C11—C17—O8	161.97 (15)
N3A—C4A—C5A—C6A	−175.9 (13)	C16—C11—C17—O8	−18.0 (2)
C4A—C5A—C6A—C1A	0.0	C12—C11—C17—O7	−18.5 (2)
C2A—C1A—C6A—C5A	0.0	C16—C11—C17—O7	161.58 (15)

### 4-(4-Nitrophenyl)piperazin-1-ium 3,5-dinitrobenzoate (3) Hydrogen-bond geometry (Å, º)

**Table:**

*D*—H···*A*	*D*—H	H···*A*	*D*···*A*	*D*—H···*A*
N2—H21···O8	0.95 (2)	1.77 (2)	2.705 (3)	170.7 (17)
N2—H22···O7^i^	0.92 (2)	1.81 (2)	2.715 (3)	166.4 (18)
C8—H8*A*···O4^ii^	0.97	2.54	3.245 (3)	130
C8—H8*B*···O2^iii^	0.97	2.40	3.354 (4)	168
C8—H8*B*···O2*A*^iii^	0.97	2.39	3.353 (16)	172
C9—H9*A*···O1^iii^	0.97	2.54	3.497 (5)	171
C9—H9*A*···O1*A*^iii^	0.97	2.47	3.378 (14)	155
C10—H10*B*···O4^iv^	0.97	2.60	3.425 (3)	143
C12—H12···O2^v^	0.93	2.53	3.231 (4)	133

Symmetry codes: (i) −*x*, −*y*, −*z*+1; (ii) −*x*−1/2, *y*−1/2, −*z*+1/2; (iii) −*x*+1/2, −*y*+1/2, −*z*+1; (iv) −*x*−1/2, *y*+1/2, −*z*+1/2; (v) *x*−1/2, *y*−1/2, *z*.
